# SimBinder-IF: structure-aware antibody affinity optimization via efficient preference learning

**DOI:** 10.1093/bioinformatics/btag548

**Published:** 2026-07-24

**Authors:** Xinyan Zhao, Yi-Ching Tang, Rivaaj Monsia, Victor J Cantu, Ashwin Kumar Ramesh, Siyu Yang, Xiaozhong Liu, Zhiqiang An, Xiaoqian Jiang, Yejin Kim

**Affiliations:** McWilliams School of Biomedical Informatics, University of Texas Health Science Center at Houston, Houston, TX 77030, United States; McWilliams School of Biomedical Informatics, University of Texas Health Science Center at Houston, Houston, TX 77030, United States; Department of Computer Science, The University of Texas at Austin, Austin, TX 78712, United States; McWilliams School of Biomedical Informatics, University of Texas Health Science Center at Houston, Houston, TX 77030, United States; Texas Therapeutics Institute, Brown Foundation Institute of Molecular Medicine, University of Texas Health Science Center at Houston, Houston, TX 77030, United States; McWilliams School of Biomedical Informatics, University of Texas Health Science Center at Houston, Houston, TX 77030, United States; Computer Science Department, Worcester Polytechnic Institute, Worcester, MA 01609, United States; Texas Therapeutics Institute, Brown Foundation Institute of Molecular Medicine, University of Texas Health Science Center at Houston, Houston, TX 77030, United States; McWilliams School of Biomedical Informatics, University of Texas Health Science Center at Houston, Houston, TX 77030, United States; McWilliams School of Biomedical Informatics, University of Texas Health Science Center at Houston, Houston, TX 77030, United States

## Abstract

**Motivation:**

Antibody therapeutic efficacy depends on high-affinity target engagement, yet laboratory affinity maturation is slow and costly. Most protein language models (PLMs) lack explicit training for high affinity, and current preference optimization methods introduce computational overhead without clear affinity improvements. Therefore, structure-aware and parameter-efficient approaches for antibody affinity optimization are urgently needed.

**Results:**

We propose SimBinder-IF, a structure-aware antibody optimization model trained by freezing the Evolutionary Scale Modeling inverse folding (ESM-IF) structure encoder and fine-tuning only its decoder via Simple Preference Optimization (SimPO) to prefer stronger binders. In generalization tests across seven held-out complexes (93 477 mutants), SimBinder-IF shows a 22% numerical increase in average Spearman correlation (0.26 to 0.32) compared with vanilla ESM-IF and outperforms ESM-IF on five of seven assays; however, the assay-level paired Wilcoxon test does not reach statistical significance (p=0.188). For seed-averaged top-ranking enrichment, SimBinder-IF has the highest 20-fold improvement@20 mean (0.453), whereas ESM-IF has the highest 10-fold improvement@10 mean (0.520 vs. 0.487 for SimBinder-IF). In a case study redesigning antibody F045-092 to target the A/California/04/2009 pandemic H1N1 (pdmH1N1) strain—a target the original antibody fails to bind—SimBinder-IF generates variants with markedly lower predicted binding free energy than ESM-IF (mean ΔΔG: −68.56 vs. −49.77 kcal/mol). Notably, SimBinder-IF updates only 18% of the ESM-IF parameters. Regarding generic protein–protein affinity modelling, SimBinder-IF shows class-dependent preservation with trade-offs, improving performance on antibody–antigen (AB/AG) and protease–protein inhibitor (Pr/PI) complexes while yielding slightly lower aggregate mean correlations than ESM-IF and ProteinDPO.

**Availability and implementation:**

The source code and model weights for SimBinder-IF are available at https://github.com/MSBMI-SAFE/SimBinder-IF.

## 1 Introduction

From the approval of the first monoclonal antibody, Muromonab‐CD3, in 1986 to the end of 2024, the U.S. Food and Drug Administration (FDA) had cumulatively authorized over 160 monoclonal antibodies (Wang *et al*. 2025). In 2024 alone, 13 new antibodies were approved, setting a record for annual approvals (de la Torre and Albericio 2025). The binding affinity between an antibody and its antigen is a principal determinant of an antibody’s therapeutic potential. Higher-affinity variants typically result in enhanced in vivo potency, reduced dosing frequency, consequently mitigating immunogenicity risk and decreasing the likelihood of immune escape (Dall’Acqua *et al*. 2006, Dyson *et al*. 2020, Muecksch *et al*. 2021). Conversely, progress with candidates that show sub-optimal affinities are frequently discontinued in pre-clinical or early clinical stages despite otherwise favourable profiles. Although phage display enables high-throughput library screening, downstream validation—such as surface plasmon resonance (SPR) or biolayer interferometry (BLI)—remains low-throughput and costly. Moreover, the method itself can introduce bias towards highly expressed or stable clones. Machine learning-based antibody design approaches complement these experimental techniques in their potential for navigating the vast search space and discovering high-affinity antibody variants that may not be readily accessible through experimental approaches alone (Shanker *et al*. 2024, Pacesa *et al*. 2025, Zhu *et al*. 2025). A computational model that can directly design candidate antibody sequences with enriched affinity holds the promise of dramatically accelerating lead discovery and reducing experimental burden.

Self-supervised protein language models (PLMs) such as the Evolutionary Scale Modeling (ESM) (Hsu *et al*. 2022, Lin *et al*. 2023, Hayes *et al*. 2025) series have demonstrated their ability to learn fundamental ‘protein grammar’; they are capable of capturing secondary and tertiary structural features and facilitating the inference of long-range residue–residue contacts (Lin *et al*. 2023, Zhang *et al*. 2024). Antibodies constitute a distinct protein class with a modular $H_{2}L_{2}$ architecture and discrete variable and constant domains (Edelman 1973, McConnell and Casadevall 2025). Diversity concentrated in six complementarity-determining regions (CDRs)—sculpted by somatic hypermutation and clonal selection—confers epitope-level specificity and affinity, distinguishing antibodies from most other proteins. However, naturally evolved proteins do not optimize solely for binding affinity, and most general PLMs—trained by self-supervision on large-scale protein databases—lack any explicit alignment with antibody–antigen binding affinity. Moreover, antibody–antigen complexes constitute less than 3% of the Protein Data Bank (PDB) (Zhou *et al*. 2025), so affinity-relevant interface signals are vastly diluted during pre-training. Furthermore, the PDB contains only complexes with sufficiently high affinity to enable structure determination, including antibody–antigen and other protein–protein complexes. As a result, PLMs and other types of protein models achieve only modest Spearman correlations (ρ≈0.1-0.5) with real in vitro affinity values on public benchmarks (Zhao *et al*. 2025). This indicates that the current PLMs struggle to design antibodies with high affinity.

Recent work has explored parameter-efficient fine-tuning (Sledzieski *et al*. 2024) and structural integration (Huang *et al*. 2023, Wee *et al*. 2024, Xu *et al*. 2025) to adapt protein models for downstream tasks. In the antibody domain, advances in CDR structure prediction (Abanades *et al*. 2022, Jeon and Kim 2024, Wei *et al*. 2026) and antibody-specific language models (Olsen *et al*. 2024) have improved structural accuracy and sequence quality, while methods targeting developability (Rollins *et al*. 2024) or embedding representations (Singh *et al*. 2025) address complementary properties. However, these approaches either focus on structural accuracy without explicit affinity optimization or target properties other than binding affinity.

Preference optimization techniques have been introduced to align models with desired properties. ProteinDPO (Widatalla *et al*. 2024) and ResiDPO (Xue *et al*. 2025) apply Direct Preference Optimization (DPO) (Rafailov *et al*. 2023) with stability-based fitness functions, focusing on global stability rather than binding affinity. Multi-Objective Binder DPO (Mistani and Mysore 2024) and CtrlProt (Liu *et al*. 2025) target receptor–peptide binding or controllable generation but rely on sequence-only architectures lacking explicit 3D structural context. While AbDPO (Zhou *et al*. 2024) formulates antibody design as a preference problem using biophysical energy, these approaches share critical limitations: they often align with proxies (e.g. stability or total energy) rather than true antibody-antigen affinity; they typically employ full-parameter fine-tuning, which risks undermining the pre-trained model’s foundational understanding; and standard DPO introduces a discrepancy between training and inference. Moreover, AbBiBench (Zhao *et al*. 2025) evaluation shows that continual training on antibody-specific datasets (CurrAb (Burbach and Briney 2025), AntiFold (Høie *et al*. 2025)) or training from scratch (Diffab (Luo *et al*. 2022), dyMEAN (Kong *et al*. 2023)) does not improve performance on antibody design tasks. These findings motivate us to rethink the training paradigm for antibody-specific models.

Evolutionary Scale Modeling inverse folding (ESM-IF) (Hsu *et al*. 2022) is a PLM originally designed for the inverse folding task, where the goal is to generate the protein sequence based on its structure. Given its state-of-the-art performance on antibody binding affinity tasks (Zhao *et al*. 2025), we adopt ESM-IF as our base model and introduce SimBinder-IF, a structure-aware antibody model trained via efficient Simple Preference Optimization (SimPO) (Meng *et al*. 2024) to generate antibodies with improved binding affinity (Fig. 1). SimBinder-IF is trained on in vitro antibody binding affinity data by encouraging sequences with higher affinity to receive higher log-likelihoods than those with lower affinity. In addition, to maintain the model’s ability to interpret protein structure, we keep the structure encoder of the base model fixed and update only the sequence decoder during fine-tuning.

**Figure 1:**
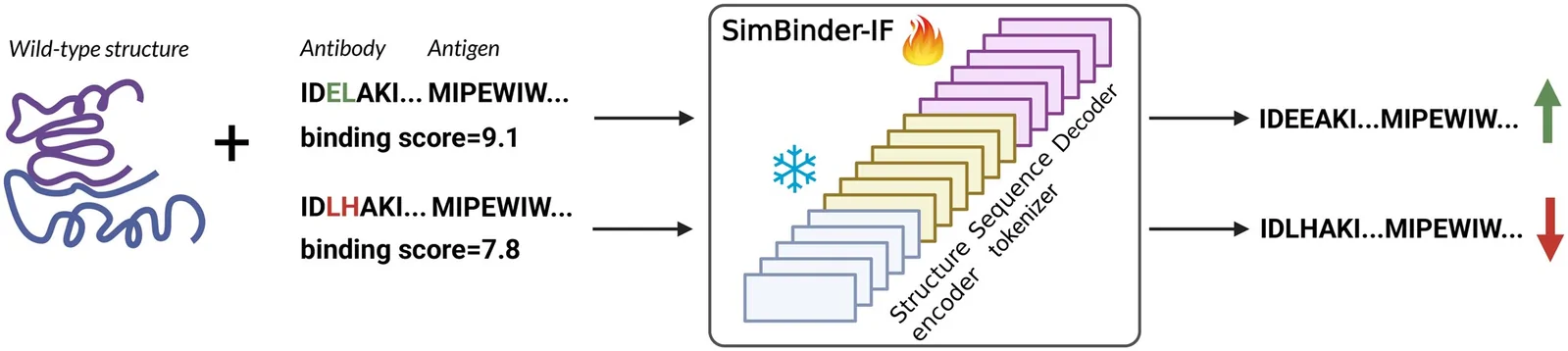
Overview of the training flow of SimBinder-IF. The model utilizes a frozen structure encoder and a trainable sequence decoder. SimBinder-IF is trained using Simple Preference Optimization (SimPO) on paired antibody-antigen sequences and structures to assign higher log-likelihoods to sequences with better binding affinity.

**Figure 2:**
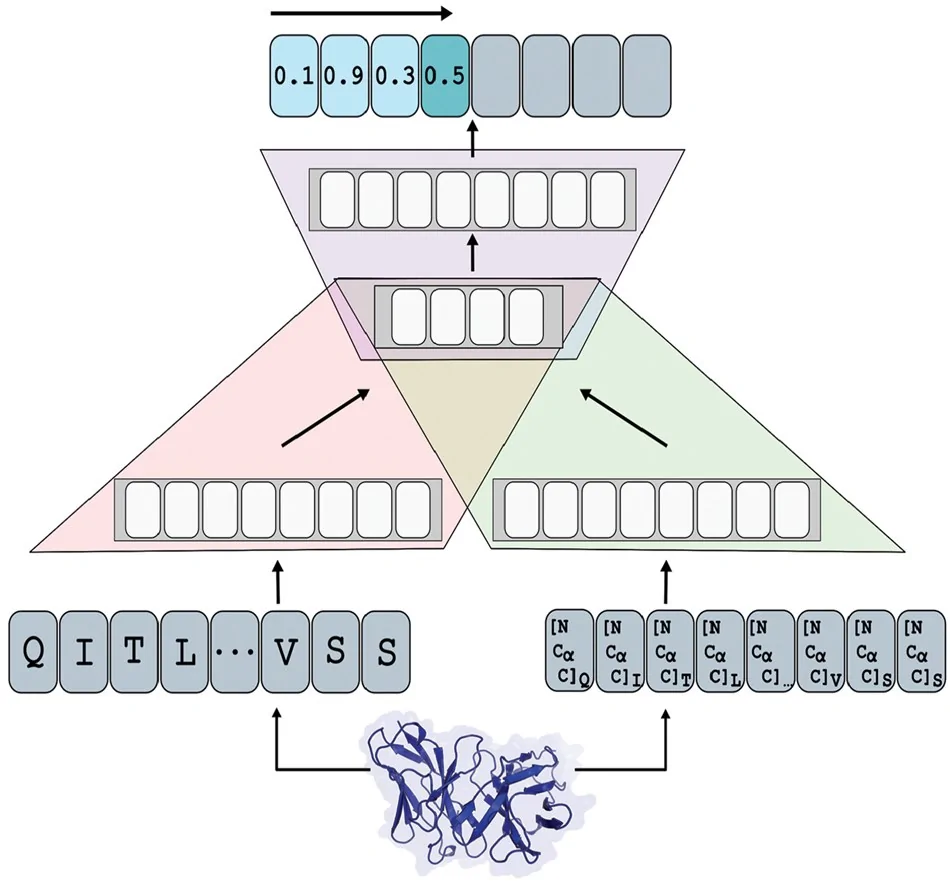
ESM-IF architecture used in SimBinder-IF. Amino acid sequence tokens (red) and geometric features extracted from the protein backbone by a structure encoder (green) are integrated by an autoregressive decoder (purple), which predicts amino acid probabilities conditioned on the backbone structure and preceding residues. SimBinder-IF applies this architecture to antibody–antigen complexes. Reproduced from Fig. 1B of Shanker et al., Science, doi: 10.1126/science.adk8946 (2024), AAAS, with permission.

Our experiments on the AbBiBench benchmark (Zhao *et al*. 2025) validated the effectiveness of SimBinder-IF model. In generalization tests on mutational datasets across seven held-out antigen–antibody complexes, with total mutants 93 477 SimBinder-IF shows a 22% numerical increase in the average Spearman correlation (from 0.26 to 0.32) compared with vanilla ESM-IF and outperforms ESM-IF on five of seven assays and has the highest seed-averaged 20-fold improvement@20 mean. Additionally, we demonstrate the model’s capability in capturing interface-specific binding signals on the m396 SARS-CoV-2 dataset. On SKEMPI 2.0, generic protein–protein interaction modelling is preserved in a class-dependent manner, with gains for antibody–antigen (AB/AG) and protease–protein inhibitor (Pr/PI) complexes but trade-offs for T-cell receptor/peptide–major histocompatibility complex (TCR/pMHC) and heterogeneous ‘Other’ complexes. To further validate SimBinder-IF, we conducted a case study redesigning the antibody F045-092 to target the A/California/04/2009 pandemic H1N1 (pdmH1N1) strain, for which the original F045-092 exhibits little to no binding affinity. Antibody mutants designed by SimBinder-IF and other models were ranked based on their predicted structural integrity and the biophysical properties of the resulting antibody–antigen complexes. SimBinder-IF outperforms other baselines in terms of predicted binding free energy (ΔΔG). Moreover, antibodies generated by SimBinder-IF exhibit the best or comparable sequence plausibility and structural credibility compared to the other models tested. These results show that our preference learning framework markedly improves the base model’s capacity to design antibody candidates with strong theoretical binding profiles, subject to future experimental validation.

## 2 Materials and methods

### 2.1 Overview of SimBinder-IF

The architecture of SimBinder-IF is shown in Fig. 2. As illustrated in Fig. 1, we fine-tune a pretrained ESM-IF (Hsu *et al*. 2022) model by freezing its structure encoder and retraining only the sequence decoder. The sequence tokenizer functions as a dictionary to convert text sequences into numerical tokens and is not updated during this process. By freezing the structure encoder, we preserve its foundational ability to capture intricate structural features of antibody–antigen complexes, prevent catastrophic forgetting of pretrained knowledge, and reduce computational overhead. The model ingests the three-dimensional structure of the wild-type antibody–antigen complex together with the sequence of a mutated antibody concatenated with its corresponding antigen sequence, and outputs the log-likelihood of the mutated antibody–antigen sequence conditioned on the structural context. To ensure that higher likelihoods correspond to stronger binding, we employ Simple Preference Optimization (SimPO) (Meng *et al*. 2024): during training, paired high and low affinity variants are presented simultaneously, and the decoder is optimized to increase the score of experimentally validated high affinity sequences while decreasing that of low affinity ones. The finetuned model demonstrates improved correlation with binding affinity in both zero-shot and supervised benchmarks and can generate novel CDR sequences predicted to enhance antigen binding.

### 2.2 Simple preference optimization for affinity

We fine-tune ESM-IF to assign higher average log-likelihoods to antibody-antigen complexes with greater binding affinity. A natural objective is Direct Preference Optimization (DPO) (Rafailov *et al*. 2023), which learns from pairwise preferences via rewards defined as $r_{\text{DPO}}(x,y)=\beta [\text{log}\;\pi_{\theta }(y|x)-\text{log}\;\pi_{\text{ref}}(y|x)]$. The loss is $L_{\text{DPO}}=-\text{log}\;\sigma (r_{\text{DPO}}(x,y_{w})-r_{\text{DPO}}(x,y_{\ell }))$. However, DPO has two key limitations for our task. First, its reward depends on a reference model $\pi_{\text{ref}}$, whereas inference relies solely on $\pi_{\theta }$. This train-test mismatch can favor sequences simply because the reference rates them poorly, leading to inconsistent likelihood ordering (Meng *et al*. 2024). Second, computing the reference policy doubles memory usage and training time.

To address this, we employ SimPO (Meng *et al*. 2024), which aligns the training objective with the generation metric by removing the reference model. SimPO defines the reward as the length-normalized log-likelihood:


$$r_{\text{SimPO}}(x,y)=\frac{\beta }{\left|y\right|}\sum_{i=1}^{\left|y\right|}\;\text{log}\;\pi_{\theta }\left(y_{i}|x,y_{<i}\right),$$

Using a Bradley-Terry model with a margin γ, the loss becomes


$$L_{\text{SimPO}}=-\text{log}\;\sigma \left(r_{\text{SimPO}}(x,y_{w})-r_{\text{SimPO}}(x,y_{\ell })-\gamma \right).$$

This approach directly optimizes the likelihood of preferred sequences, eliminating spurious score inflation and reducing memory and computational costs by avoiding the reference pass.

### 2.3 Log-likelihood as a proxy for antibody–antigen binding affinity

Prior efforts in protein fitness benchmarking demonstrate that model likelihood correlates with functional performance. For example, ProteinGym (Notin *et al*. 2023) measures correlations between perplexity and general fitness, while FLAb (Chungyoun *et al*. 2024) extends this framework to antibodies by compiling affinity and developability data.

Shanker *et al*. (2024) found that the log-likelihood of antibody sequences conditioned on antigen-bound backbones (using ESM-IF) correlates strongly with binding affinities across mutational datasets. Following this, AbBiBench (Zhao *et al*. 2025) treats the Ab–Ag complex as the input for zero-shot prediction. It computes the Spearman correlation between model log-likelihood and experimental binding affinity. A high correlation indicates that stronger binders receive higher likelihoods, supporting the use of log-likelihood for affinity ranking.

### 2.4 Two-stage screening pipeline for antibody optimization

We follow the AbBiBench protocol (Zhao *et al*. 2025) to redesign the F045-092 CDR-H3 loop. For each model, we sample 1500 sequences (up to five mutations) conditioned on the AlphaFold 3 predicted complex structure.

In Stage 1 (Rapid Prescreen), candidates are independently ranked by sequence naturalness (AntiBERTy (Ruffolo *et al*. 2021) log-likelihood) and predicted binding affinity (FoldX ΔΔG). Only the variants scoring in the top 30% across both metrics simultaneously advance to Stage 2. In Stage 2 (High-Confidence Ranking), candidates are re-folded with AlphaFold 3 (Abramson *et al*. 2024) and the final selection identifies Pareto-optimal variants across six objectives: FoldX ΔΔG, ΔEpitope SASA, AntiBERTy log-likelihood, interface predicted Template Modelling score (iPTM), interface predicted Local Distance Difference Test (ipLDDT), and ProteinMPNN (Dauparas *et al*. 2022) log-likelihood.

### 2.5 Experimental datasets

#### 2.5.1 AbBiBench for training and evaluating Ab-Ag binding affinity

To train and test the efficacy of the proposed method, we utilize the AbBiBench dataset, which includes compiled data for 13 antibody-antigen binding-affinity assays (Table 1). Each assay includes curated data on the heavy chain or light chain sequence, experimental binding affinity measurements, and wild-type antibody-antigen complex structure. Each assay ranges from 419 to 65 535 mutants. Moreover, experimental binding affinities were standardized from *K_d_* values (dissociation constants) and enrichment ratios to binding scores via transformations using negative log and log, respectively. In turn, a higher binding score denotes more favourable binding across the entire dataset. Evaluation on AbBiBench is stratified according to each antigen-antibody assay to ensure a fair, accurate assessment over a range of structures.

**Table 1:** Overview of the 13 Ab-Ag binding-affinity assays collected by AbBiBench (Zhao *et al.* 2025).

ID	Antibody	Antigen	Source	Variants	Binding score	Study
Training set						
4fqi_h1	CR9114	Influenza A/New Caledonia/20/99 (H1N1).	Human IgG1	HC: 65 094	$-\text{log}\;K_{d}$	(Phillips *et al.* 2021)
3gbn_h1	CR6261	Influenza A/New Caledonia/20/99 (H1N1).	Human IgG1	HC: 1887	$-\text{log}\;K_{d}$	(Phillips *et al.* 2021)
aayl51	AAYL51	Spike HR2	Human IgG1	HC: 4320	$-\text{log}\;K_{d}$	(Engelhart *et al.* 2022)
aayl52	AAYL52	Spike HR2	Human IgG1	LC: 13 324	$-\text{log}\;K_{d}$	(Engelhart *et al.* 2022)
2fjg	G6.31	VEGF	Human IgG1	HC: 2223 LC: 2014.	*log* enrichment	(Koenig *et al.* 2017)
1mlc	D44.1	Hen-egg-white lysozyme	Mouse IgG1	HC: 1229 LC: 865.	*log* enrichment	(Warszawski *et al.* 2020)
Test set (held-out complexes)						
4fqi_h3	CR9114	Influenza A/Wisconsin/67/2005 (H3N2).	Human IgG1	HC: 65 535	$-\text{log}\;K_{d}$	(Phillips *et al.* 2021)
3gbn_h9	CR6261	Influenza A/Hong Kong/1073/1999 (H9N2).	Human IgG1	HC: 1842	$-\text{log}\;K_{d}$	(Phillips *et al.* 2021)
aayl49	AAYL49	Spike HR2	Human IgG1	HC: 4312	$-\text{log}\;K_{d}$	(Engelhart *et al.* 2022)
aayl49_ML	AAYL49_ML	Spike HR2	Human IgG1	HC: 8953	$-\text{log}\;K_{d}$	(Li *et al.* 2023)
aayl50	AAYL50	Spike HR2	Human IgG1	LC: 11 473	$-\text{log}\;K_{d}$	(Engelhart *et al.* 2022)
1n8z	trastuzumab	HER2	Humanized IgG1	HC: 419	$-\text{log}\;K_{d}$	(Shanehsazzadeh *et al.* 2023)
5a12_ang2	5A12	Ang2	Human IgG1	HC: 796 LC: 104 Both: 43.	*log* enrichment	(Minot and Reddy 2024)

Assays are split into a training set and a held-out test set at the complex level.

We appraise the performance of the model on a rigorous zero-shot setting. We reserved seven antigen–antibody mutation datasets as our test set to ensure broad, biologically meaningful evaluation:

- 1n8z (trastuzumab–HER2) tests a clinically established therapeutic antibody targeting a medically relevant oncological epitope.
- 3gbn_h9 (CR6261–Influenza A/H9N2) and 4fqi_h3 (CR9114–Influenza A/H3N2) challenge mutational modelling in rapidly evolving viral proteins.
- aayl49 (AAYL49–SARS-CoV-2 Spike HR2) tests generalization to the coronavirus HR2 fusion helix, assessing transfer across viral families and epitope geometries.
- aayl49_ML (AAYL49_ML–SARS-CoV-2 Spike HR2) induces distribution shift via an ML-designed phage-display library, testing robustness to algorithmically proposed sequences.
- aayl50 (AAYL50–SARS-CoV-2 Spike HR2) evaluates a different antibody scaffold against the same antigen, testing alternative paratope solutions and chain-specific effects (HC vs LC).
- 5a12_ang2 (5A12–Ang2) evaluates a clinically relevant human target with enrichment-style readouts, testing whether the likelihood–affinity link transfers beyond viral antigens.

To verify the rigor of this antigen-split evaluation, we computed pairwise sequence identities between all training and test complexes (Fig. S2, available as supplementary data at *Bioinformatics* online). Antigen sequence identity is below 4% for the vast majority of training–test pairs, confirming that the model faces antigens with no close sequence homologs in the training set at test time. Antibody (VH + VL) sequence identity across non-scaffold-shared pairs averages ≈53.2%, consistent with natural framework region conservation. For 3gbn_h9 and 4fqi_h3, the antibody–antigen structure is shared with the corresponding training assays (3gbn_h1 and 4fqi_h1), yet binding score Spearman correlations across hemagglutinin subtypes are only ρ=0.725 (H1→H9) and ρ=0.379 (H1→H3), confirming that these pairs pose a genuine cross-subtype generalization challenge rather than mere memorization.

#### 2.5.2 m396 SARS-CoV-2 antibody re-engineering in silico mutational dataset

To assess SimBinder-IF’s ability to model both global and interface-specific binding in antibody–antigen complexes, we adopt the large-scale in silico mutational dataset from Desautels *et al*. (2020) on m396 antibody re-engineering. The m396 antibody, a potent SARS-CoV-1 neutralizer, exhibits insufficient affinity towards the SARS-CoV-2 RBD, motivating computational redesign.

The dataset contains 89 263 distinct variants generated through iterative mutational scanning of the m396 sequence on homology-based structural models. For each mutant, FoldX-based free-energy estimates are reported for both (i) whole-complex stability changes and (ii) interface-restricted binding free-energy changes that isolate contributions from the RBD–antibody contact region. We use these FoldX whole-complex and interface-only ΔΔG values as proxy labels to evaluate model performance via correlation metrics and top-rank precision.

#### 2.5.3 SKEMPI 2.0 for generic protein-protein binding affinity evaluation

We use SKEMPI 2.0 (Jankauskaitė *et al*. 2019) as the benchmark dataset to assess the ability to model generic protein–protein binding affinity. Each entry contains the PDB complex identifier, mutation annotations, and equilibrium dissociation constants for mutant and wild type. After discarding entries with missing or non-numeric affinity values, we convert dissociation constants into ΔΔG labels in kcal/mol using $\Delta \Delta G=RT\text{ln}(K_{d}^{\text{mut}}/K_{d}^{\text{wt}})$ with $R=1.9872\times 10^{-3}$ kcal·mol${\;}^{-1}\cdot$K${\;}^{-1}$. For each complex, we apply the specified mutations to the amino-acid sequences while keeping backbone coordinates fixed, generating mutant and wild-type multi-chain sequences that are concatenated as model inputs. For per-complex correlation analysis, we retain only complexes with at least 200 mutation entries and non-zero variance in experimental ΔΔG to ensure stable Spearman correlation estimates.

#### 2.5.4 Case study: F045-092 antibody redesign against pdmH1N1 influenza

To evaluate the capacity of protein language models to design antibody variants with enhanced binding towards a given antigen, we performed a case study centred on F045-092 (Ohshima *et al*. 2011). This antibody was discovered through comprehensive screening of a human B-cell repertoire using phage-display technology. While initially raised against H3 influenza virus particles, this antibody also demonstrated a broad range of binding activity against different strains belonging to H1N1, H2N2 and H5N1 viruses (Ohshima *et al*. 2011). However, F045-092 shows little to no binding towards A/California/04/2009 (pdmH1N1) strain. This has been attributed to a steric bulge present in all H1N1 strains that emerged after 2009, caused by the insertion of a lysine residue at position 133a (Ekiert *et al*. 2012, Simmons *et al*. 2023). No experimentally solved structure of the F045/pdmH1N1 complex has been reported to date, and this antibody–antigen pair has not been previously explored in computational antibody design literature. Therefore, our study constitutes a fully novel evaluation setting, ensuring the absence of training–test data leakage. The structure of the F045/pdmH1N1 complex used in this study was generated using AlphaFold 3 (Abramson *et al*. 2024).

### 2.6 Training data construction and parameter efficiency

#### 2.6.1 Training data construction and balancing

Within each assay batch, samples are grouped by parent wild-type complex, shuffled, and paired via cyclic right-shift (each mutation paired with the next in the shuffled list), so no sample is paired with itself. If $\text{affinity}(x_{i})>\text{affinity}(x_{j})$, $\left(x_{i},x_{j}\right)$ becomes a (chosen, rejected) pair for the SimPO loss; otherwise roles are reversed. This avoids exhaustive enumeration while sampling pairs uniformly across each batch.

Training assays span 1229–65 094 mutations. All mutations are pooled into a single globally shuffled dataset, so gradient updates naturally downsample large assays without discarding data. For model selection, 20% of mutations per training complex are reserved as validation; the checkpoint with the highest average Spearman ρ across validation assays, evaluated every 100 steps, is retained.

#### 2.6.2 Parameter efficiency

We freeze the geometric vector perceptron graph neural network (GVP–GNN) encoder while training only the decoder, reducing the number of trainable parameters to roughly 25 M (about 18% of the entire ESM-IF) without losing the geometric conditioning provided by the frozen encoders. On a single NVIDIA H200 GPU, decoder-only fine-tuning requires 12.4 h per epoch and 17.9 GB of GPU memory, compared with 15.5 h and 70.1 GB for full-parameter fine-tuning. Full hyperparameter settings are provided in Supplementary Section S4, available as supplementary data at *Bioinformatics* online.

### 2.7 Baselines

We compare SimBinder-IF against a diverse set of baselines, including general protein language models, antibody-specific models, and preference-optimized variants. This comprehensive comparison allows us to evaluate whether specialized training on antibody data and preference optimization strategies yield performance improvements over strong general-purpose backbones.

#### 2.7.1 General protein language models

We include three structure-aware or sequence-based models trained on large-scale protein databases: (i) *SaProt* (Su *et al*. 2024), a structure-aware masked language model that encodes both sequence and structure information, achieving leading performance in the AbBiBench test; (ii) *ESM-2* (Lin *et al*. 2023), a large-scale protein language model trained on sequence data that has demonstrated strong transfer learning capabilities across diverse protein tasks; and (iii) *ESM-IF* (Hsu *et al*. 2022), the base inverse folding model used in our work, which conditions sequence generation on 3D backbone geometry.

#### 2.7.2 Antibody-specific models

To assess whether domain-specific training improves antibody design, we evaluate: (i) *CurrAb* (Burbach and Briney 2025), which applies curriculum learning to fine-tune ESM-2 on antibody-specific datasets; (ii) *AntiFold* (Høie *et al*. 2025), an inverse folding model specifically trained on antibody structures from ESM-IF; (iii) *Diffab* (Luo *et al*. 2022), a diffusion-based generative model trained from scratch for antigen-specific antibody design; and (iv) *dyMEAN* (Kong *et al*. 2023), an end-to-end deep learning framework for de novo antibody design.

#### 2.7.3 Preference-optimized models

We compare against *ProteinDPO* (Widatalla *et al*. 2024), which fine-tunes ESM-IF using direct preference optimization (DPO) to favor stabilizing variants over destabilizing ones. To ensure a fair comparison and isolate the contributions of our training strategy, we also include three ESM-IF variants trained on the AbBiBench dataset: (i) ESM-IF fine-tuned with DPO while updating only the decoder parameters (ESM-IF-DPO-Dec); (ii) ESM-IF fine-tuned with SimPO across all model parameters (ESM-IF-SimPO-All); and (iii) ESM-IF fine-tuned with SimPO while updating half of the encoder layers along with the decoder (ESM-IF-SimPO-Hybrid).

## 3 Results

### 3.1 Using SimBinder-IF log-likelihood as a proxy for Ab-Ag binding affinity

We evaluated how preference fine-tuning affects the ability of ESM-IF to predict antigen-antibody binding affinities under a *held-out setting*, meaning that the training mutants and test mutants originate from completely different antibody-antigen complexes. To assess how well a model’s log-likelihood aligns with experimentally validated affinity measurements, we computed the Spearman correlation between the model-assigned likelihood and the experimentally measured binding affinity. A higher correlation indicates that the model assigns greater likelihood to stronger binders, suggesting that it can identify affinity-enhancing mutations in a zero-shot scenario.

To further evaluate how effectively the model prioritizes high-affinity variants among its top-ranked predictions, we additionally report the *10-fold improvement@10* and *20-fold improvement@20*, defined as the proportion of the top-*N* ranked variants that achieve at least an *N*-fold improvement in binding affinity relative to the wild type. The fold change was computed from the difference between the $pK_{d}$ values of the mutant and wild-type antibodies (i.e. $pK_{d}=-{\;\text{log}\;}_{10}K_{d}$), and exponentiated as


$$10^{\left(pK_{d,\text{mutant}}-pK_{d,\text{wild}-\text{type}}\right)},$$

Which yields the ratio of the dissociation constants $K_{d}$ between the wild-type and mutant antibodies.

#### 3.1.1 Overall results

The Spearman’s correlation coefficient scores of different models across different complexes, the 10-fold improvement@10 and the 20-fold improvement@20 are shown in Fig. 3. Results for SimBinder-IF and the fine-tuned ablation models are averaged across three seeds; the remaining baselines are evaluated once. Overall, SimBinder-IF achieves the highest average Spearman correlation among all baselines—including both general protein language models (SaProt (Su *et al*. 2024), ESM-2 (Lin *et al*. 2023), ESM-IF (Hsu *et al*. 2022)) and antibody-specific models (CurrAb (Burbach and Briney 2025), AntiFold (Høie *et al*. 2025), ProteinDPO (Widatalla *et al*. 2024), Diffab (Luo *et al*. 2022) and dyMEAN (Kong *et al*. 2023)). For the seed-averaged per-assay mean across the five $K_{d}$-based assays, SimBinder-IF has the highest 20-fold improvement@20 mean (0.453 vs. 0.430 for ESM-IF), whereas ESM-IF has the highest 10-fold improvement@10 mean (0.520 vs. 0.487 for SimBinder-IF).

**Figure 3:**
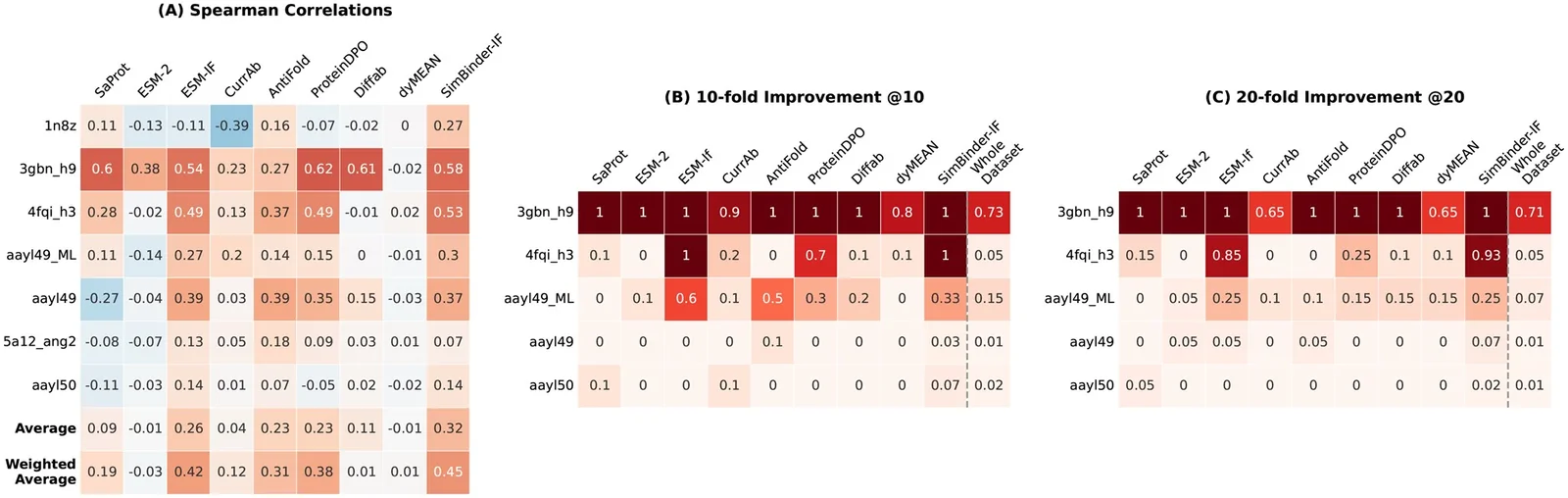
Performance comparison on the AbBiBench dataset under the zero-shot setting. (A) Spearman correlations between model-predicted likelihoods and experimentally measured binding affinity. (B) The 10-fold improvement@10 metric. (C) The 20-fold improvement@20 metric. Values for SimBinder-IF are averages across three random seeds; the ‘Whole Dataset’ column reports assay-level prevalence and is not used for model comparison.

Interestingly, the results further indicate that antibody-specific models do not automatically outperform general protein models. Both (i) fine-tuning general models on antibody data (CurrAb, AntiFold and ProteinDPO) and (ii) training antibody models from scratch (Diffab and dyMEAN) fail to yield performance gains over strong general-purpose backbones. This observation highlights the non-trivial nature of transferring protein LLMs to antibody–antigen binding prediction, and underscores the value of our proposed approach.

Compared with ESM-IF, SimBinder-IF improves overall mutant affinity ranking and the 20-fold improvement@20 metric, whereas ESM-IF retains a higher 10-fold improvement@10 mean. The gains are most pronounced on the 20-fold improvement@20 metric, where SimBinder-IF prioritizes more affinity-enhancing variants than ESM-IF. These results suggest that our proposed training paradigm improves the model’s capacity to capture subtle mutation–affinity signals, although the relative top-ranking performance depends on the predefined affinity-improvement threshold (10-fold versus 20-fold).

To assess reliability, we computed 95% bootstrap confidence intervals (CIs) for the Spearman correlations across the seven assays (Supplementary Section S2, available as supplementary data at *Bioinformatics* online). Based on the three-seed averages, SimBinder-IF shows a numerical mean improvement over ESM-IF and outperforms it on five of seven assays. The two lower-scoring assays are aayl49 (Δρ=−0.018) and 5a12_ang2 (Δρ=−0.059). SimBinder-IF also shows a mean Δρ of +0.098 relative to ProteinDPO.

#### 3.1.2 Ablation study and mechanism analysis

##### 3.1.2.1 Ablation study

In the ablation study, we compare four training strategies (with all quantitative results being the average of three seeds): (i) full fine-tuning (ESM-IF-SimPO-All), (ii) updating half of the encoder together with the full decoder (ESM-IF-SimPO-Hybrid), and (iii) decoder-only training with DPO (ESM-IF-DPO-Dec) and (iv) decoder-only training with SimPO (SimBinder-IF). As shown in Fig. 4A, SimBinder-IF achieves the best overall performance (average ρ=0.323 ± 0.012), while expanding updates to include the encoder degrades results (ESM-IF-SimPO-Hybrid: average ρ=0.267±0.007), and full fine-tuning performs the worst (ESM-IF-SimPO-All: average ρ=0.264±0.005). This trend suggests that the pretrained structure-conditioned representations are sensitive to broad parameter updates, whereas restricting adaptation to the decoder provides a safer and more effective way to absorb the preference signal without disrupting transferable features. Moreover, replacing SimPO with DPO leads to a clear performance drop (ESM-IF-DPO-Dec: average ρ=0.293±0.007). Taken together, updating the encoder parameters leads to a larger degradation in performance. These findings demonstrate that the gains are driven by where we apply adaptation and which preference objective we use, rather than by training more parameters—highlighting an efficient and robust recipe for generalizable improvements.

**Figure 4:**
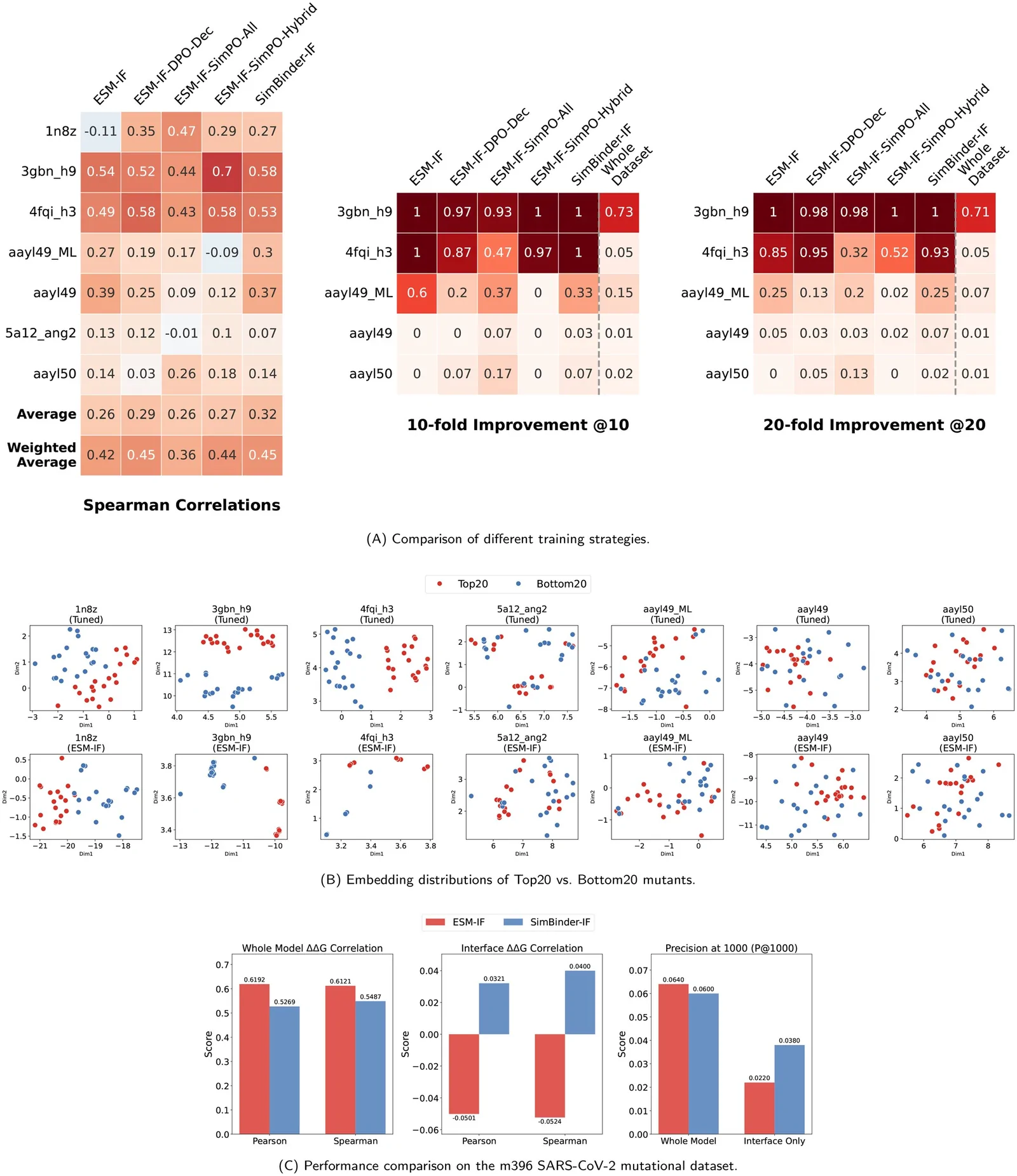
Ablation study and mechanism analysis. (A) Performance of SimBinder-IF (decoder-only SimPO) compared to full fine-tuning, hybrid updates, and DPO-based training. (B) SimBinder-IF refines affinity prediction while preserving latent structure. (C) SimBinder-IF shows superior interface modelling, indicated by positive interface ΔΔG correlation.

##### 3.1.2.2 Mechanistic insights through latent space analysis

To further investigate the impact of fine-tuning on the latent representations of antibody mutants, we visualized the embeddings of the top 20 and bottom 20 variants for each complex using t-SNE. As shown in Fig. 4B, for most datasets, the performance difference between SimBinder-IF and ESM-IF in distinguishing the highest-affinity mutants from the lowest-affinity mutants within each dataset is not pronounced. The two models exhibit broadly similar patterns of overlap and separation in the embedding space. This observation suggests that the performance gains of SimBinder-IF are not primarily derived from a radical separation of extreme samples in the representation space. Instead, the improvements are likely attributable to a more robust global modelling capability across the entire mutation landscape, as reflected in the increased Spearman correlation coefficients, and to the model’s enhanced sensitivity in prioritizing variants with substantial affinity gains, as evidenced by the improvements in the N-fold-improvement@N metrics. Furthermore, the fact that the embedding separation for top and bottom mutants remains comparable to that of the base model implies that SimBinder-IF preserves the generalizable features of the pre-trained ESM-IF and has not overfitted to the specific artefacts of the training datasets.

##### 3.1.2.3 Enhanced interface-specific generalization in the m396 SARS-CoV-2 task

To evaluate the capacity of SimBinder-IF to model binding at both the whole-complex and interface levels for antibody–antigen interactions, we adopt the large-scale in silico mutational dataset released by Desautels *et al*. (2020) on m396 antibody re-engineering during the COVID-19 pandemic. The wild-type m396 antibody was originally developed to recognize the receptor-binding domain of the SARS-CoV-1 spike protein. During the COVID-19 pandemic, Desautels *et al*. (2020) sought to re-engineer this antibody to target the RBD of the SARS-CoV-2 spike instead. To this end, they generated approximately 89 263 in silico mutant variants and estimated the binding efficacy of each candidate by computing ΔG scores using FoldX, including the FoldX Whole and FoldX Interface-Only metrics.

As illustrated in Fig. 4C, although SimBinder-IF exhibited slightly lower performance on global stability metrics (FoldX Whole Model) compared to ESM-IF, it demonstrated an improvement in predicting interface-specific effects. SimBinder-IF achieved higher precision in identifying top-ranked interface mutations (P@1000: 0.0380 vs. 0.0220) and maintained positive correlations with FoldX Interface-Only ΔΔG values, whereas ESM-IF showed negative correlations in this region. These results reveal that the competitive advantage of SimBinder-IF stems primarily from its enhanced capability to model Ab-Ag interface regions, allowing it to better capture critical binding determinants that may be diluted in global stability assessments.

### 3.2 SimBinder-IF’s ability to model generic protein–protein binding affinity

In this section, we use the SKEMPI 2.0 dataset (Jankauskaitė *et al*. 2019) to evaluate the ability of SimBinder-IF to model binding affinity for generic protein–protein complexes. For each SKEMPI entry, we compute the binding free energy ΔG of the mutant and wild type from their equilibrium dissociation constants $K_{d}$, and derive the experimental $\Delta \Delta G=\Delta G_{\text{mut}}-\Delta G_{\text{wt}}=RT\text{ln}(K_{d}^{\;\text{mut}}/K_{d}^{\text{wt}})$, which we treat as a measure of the change in binding affinity induced by the mutation. In parallel, we estimate the model-based ΔΔG as the sum of log-likelihood differences between the wild-type and mutant residues at the mutated positions:


(1) $$\Delta \Delta G_{\text{model}}=\sum_{i\in M}\;\text{log}\;\left[\frac{p_{\theta }(x_{i}^{\text{WT}}|\text{context})}{p_{\theta }(x_{i}^{\text{mut}}|\text{context})}\right],$$

Where M denotes the set of mutated sites, $x_{i}^{\text{WT}}$ and $x_{i}^{\text{mut}}$ are the wild-type and mutant residues at position *i*, and $p_{\theta }(\cdot )$ is the probability assigned by model. We aggregate mutation entries by reference complex ID and compute Spearman correlations only for complexes with at least 200 mutations and non-zero variance in experimental ΔΔG (the complete data filtering and complex grouping procedure is described in the Section 2.5.3). The filtered dataset comprises a T-cell receptor and peptide–MHC (TCR/pMHC) mutant dataset, four mutant datasets for protease–protein inhibitor (Pr/PI) complexes, and four additional mutant datasets that are neither antibody–antigen nor TCR/pMHC or Pr/PI complexes.

As shown in Fig. 5, performance on SKEMPI 2.0 is heterogeneous across complex classes. SimBinder-IF leads on antibody–antigen (AB/AG, mean ρ=0.128 vs. ESM-IF 0.106 and ProteinDPO 0.106) and Pr/PI (mean ρ=0.071 vs. ESM-IF 0.056 and ProteinDPO 0.057) complexes, which together constitute the majority of the SKEMPI 2.0 dataset by mutant count. By contrast, SimBinder-IF underperforms ProteinDPO on the TCR/pMHC subset (mean ρ=−0.003 vs. ProteinDPO 0.097, though substantially above ESM-IF −0.149) and falls below both comparators on the heterogeneous ‘Other’ category (mean ρ=0.049 vs. ESM-IF 0.122 and ProteinDPO 0.090). Across all complexes, the unweighted mean Spearman correlation of SimBinder-IF is 0.069, compared to 0.081 for ESM-IF and 0.085 for ProteinDPO; the mutant-count-weighted mean is 0.062 vs. 0.065 for ESM-IF and 0.074 for ProteinDPO. Taken together, SimBinder-IF broadly maintains, but does not uniformly improve, generic protein–protein affinity modelling: it strengthens performance on complex classes most closely related to antibody–antigen interactions, at the cost of performance trade-offs in long-tail complex classes.

**Figure 5:**
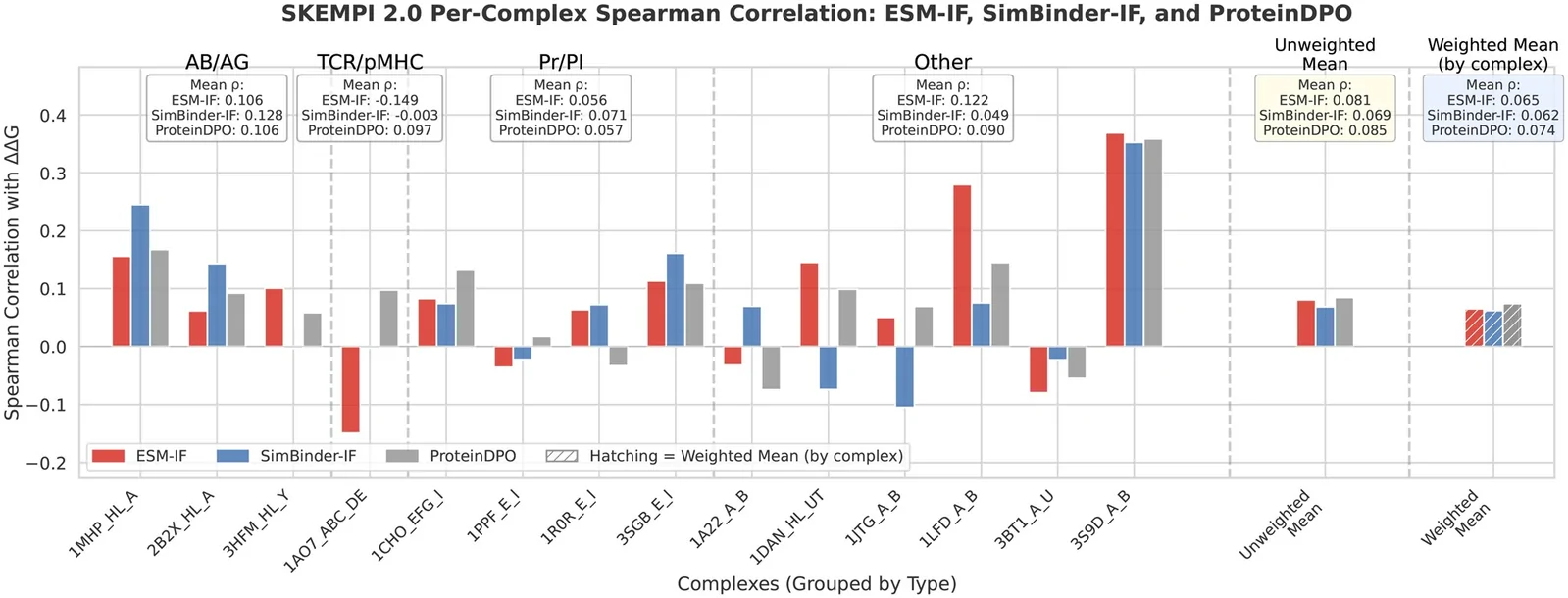
Per-complex Spearman correlation with experimental ΔΔG on the SKEMPI 2.0 dataset. Comparison among ESM-IF, SimBinder-IF, and ProteinDPO models, grouped by complex class.

### 3.3 Case study: redesign of the F045-092 antibody to target pdmH1N1 influenza hemagglutinin

To assess the capacity of PLMs on their ability to generate antibody variants with improved binding affinity towards a specific antigen, we conducted a case study on anti-influenza A antibody F045-092 (Ohshima *et al*. 2011). This antibody showed little to no binding towards A/California/04/2009 (pdmH1N1) strain. Details of this antibody are provided in Section 2.5.4. Based on the AbBiBench results, we systematically evaluated the generative capacity of our SimBinder-IF model and compared it in parallel with ESM-IF, SaProt (Su *et al*. 2024), AntiFold (Høie *et al*. 2025), and ProteinDPO (Widatalla *et al*. 2024) on their ability to redesign the CDR-H3 region of F045-092. We generated 1500 variants per model under a constraint of up to five amino acid substitutions. Following the two-stage multi-objective screening pipeline described in Section 2.4, the process distilled a Pareto-optimal panel of 15 variants: four from AntiFold, four from ESM-IF, one from ProteinDPO, and six from SimBinder-IF (SaProt yielded no Pareto-optimal candidates).

#### 3.3.1 Quantitative metrics analysis of designed variants

Detailed metric distributions across the three stages of the screening pipeline are shown in Fig. 6.

**Figure 6:**
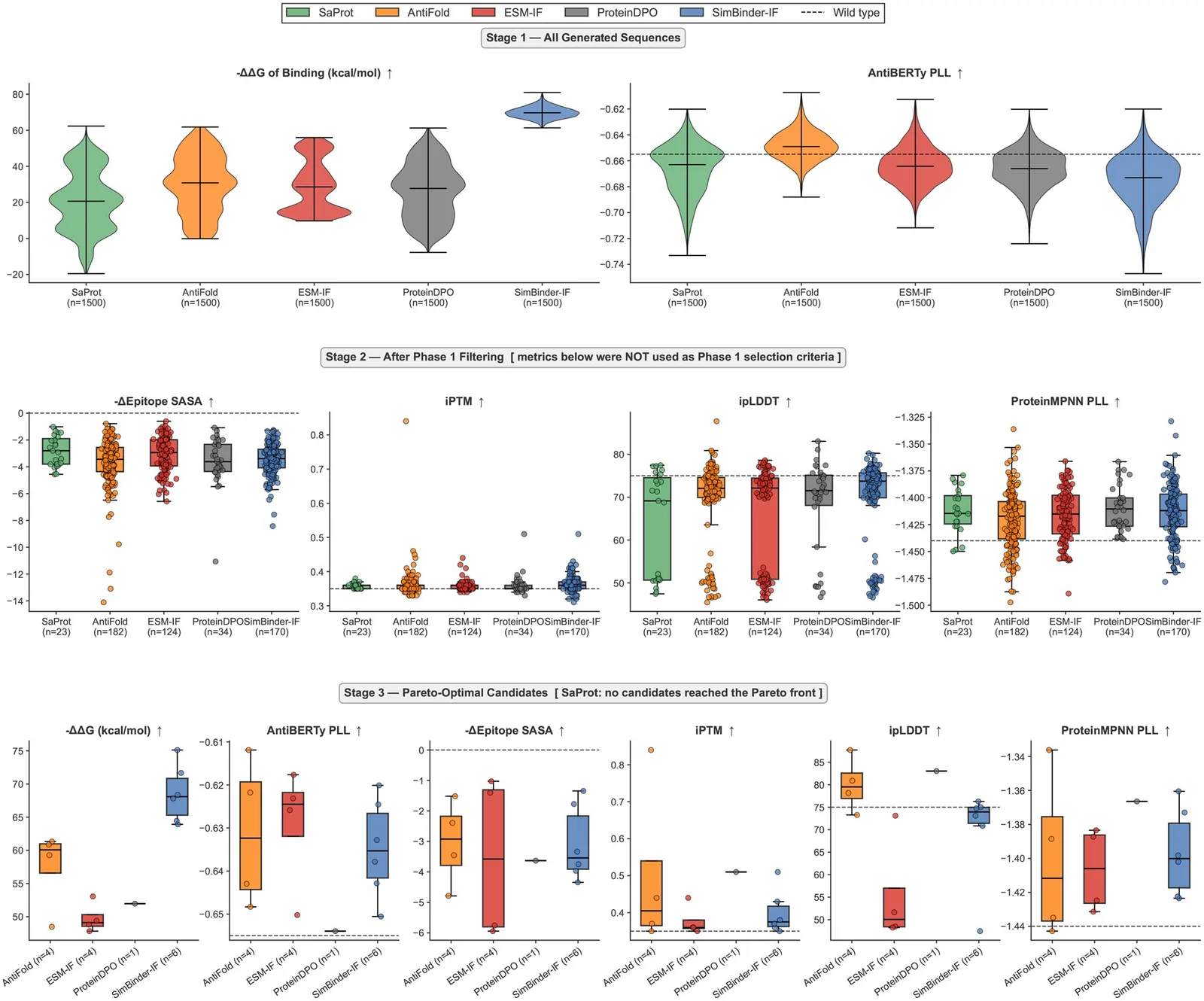
Metric distributions across the three stages of the screening pipeline. Stage 1 shows violin plots of −ΔΔG and AntiBERTy pseudo-log-likelihood (PLL) for all 1500 generated sequences per method. Stage 2 shows box plots of four structure-based metrics (−ΔEpitope SASA, iPTM, ipLDDT, ProteinMPNN PLL) for variants passing Stage 1 filtering. Stage 3 shows all six multi-objective metrics restricted to the final Pareto-optimal candidates, with SaProt excluded as it yielded zero candidates. Dashed lines indicate wild-type reference values. All metrics are oriented such that higher values represent improvement (↑).

The Stage 1 violin plots in Fig. 6 display −ΔΔG and AntiBERTy pseudo-log-likelihood (PLL) distributions for all 1500 generated sequences per method prior to any filtering. SimBinder-IF already exhibits better energy −ΔΔG distribution relative to the baselines at this pre-filtering stage. Furthermore, from the Pareto-front results, we have the following observations:

1. *Binding energy change*. SimBinder-IF achieves the lowest mean FoldX ΔΔG of −68.56 kcal/mol (n=6), outperforming AntiFold (−57.49 kcal/mol, n=4), ProteinDPO (−51.96 kcal/mol, n=1), and ESM-IF (−49.77 kcal/mol, n=4), confirming that preference fine-tuning translates into tighter predicted antigen engagement.
2. *Structure-aware quality metrics*. ProteinDPO achieves the highest mean ipLDDT (83.0) and iPTM (0.51) scores, followed closely by AntiFold (80.0 and 0.50), indicating the most favourable relative confidence in their predicted binding interfaces among the evaluated methods. SimBinder-IF substantially outperforms ESM-IF on ipLDDT (69.6 vs. 55.4) and −ΔEpitope SASA (−3.1 vs. −3.5), suggesting improved interface packing quality. All methods achieve competitive ProteinMPNN log-likelihood scores, confirming that the binding affinity advantage of SimBinder-IF is not achieved at the expense of inverse-folding plausibility.
3. *Sequence plausibility*. AntiBERTy PLL scores are comparable across methods (range: −0.629 to −0.654), indicating that the variation in binding energy and structural quality is not driven by differences in sequence naturalness.

Taken together, SimBinder-IF generates variants with the strongest predicted binding affinity among all five methods, and AntiFold and ProteinDPO achieve higher structural confidence scores, demonstrating the complementary strengths of their design preference. Antibody–antigen complex structures for all Pareto-optimal variants are presented in Section 3.3.2.

#### 3.3.2 Variant–antigen complex structures for all pareto-optimal variants

Figure 7 shows AlphaFold 3 (Abramson *et al*. 2024) predicted complexes for all Pareto-optimal variants from AntiFold, ESM-IF, ProteinDPO, and SimBinder-IF (SaProt yielded none), with the antigen as a platinum-grey surface, the heavy chain in deep crimson, and the light chain in steel blue, all aligned on the antigen.

**Figure 7:**
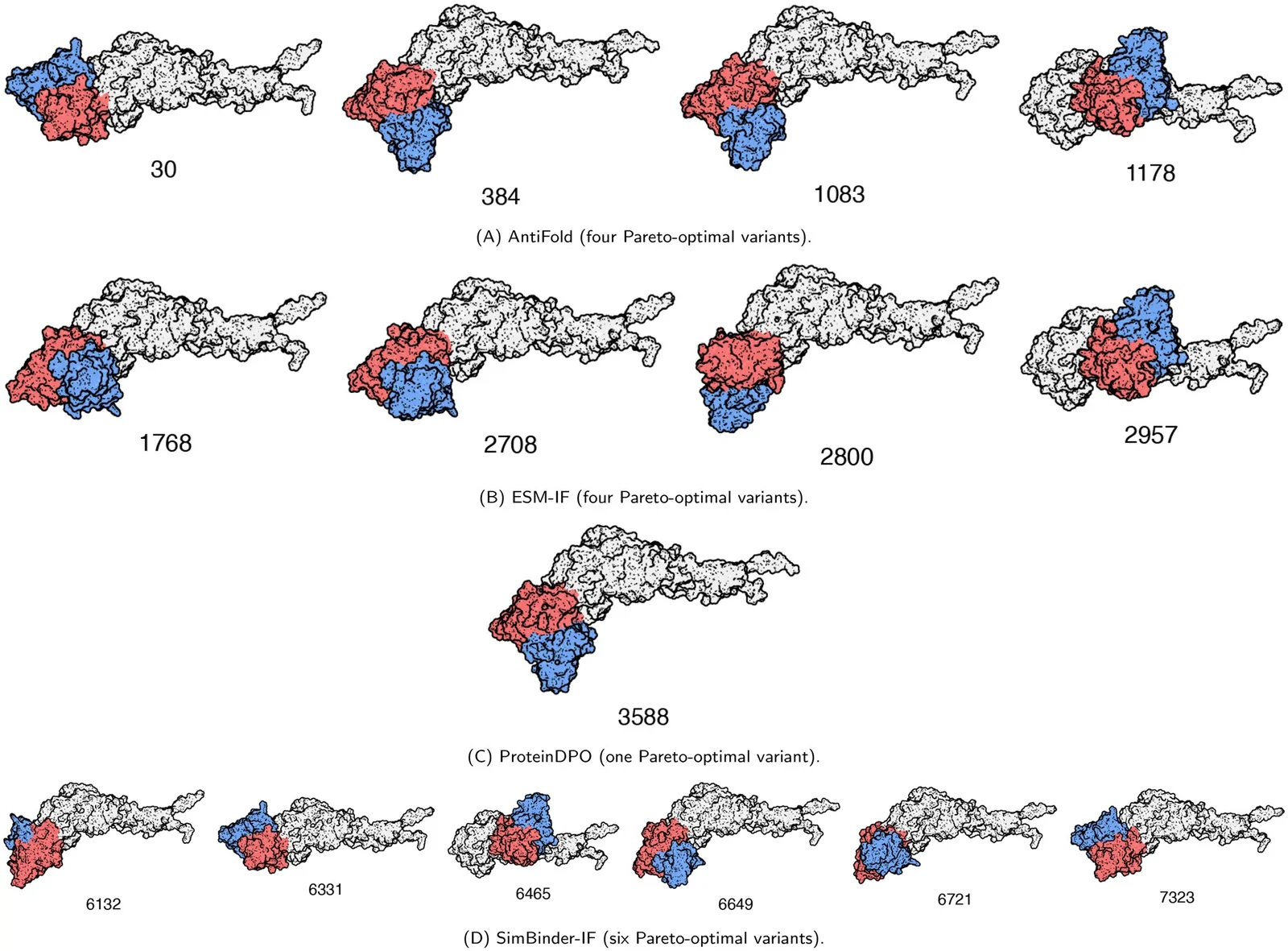
AlphaFold 3 predicted antibody–antigen complex structures for all Pareto-optimal variants. The antigen is shown as a platinum-grey surface; the antibody heavy chain as a deep-crimson surface; the light chain as a steel-blue surface. All structures within each panel are aligned on the antigen chain. SaProt generated no Pareto-optimal candidates and is not shown.

All four methods mainly target the same HA head epitope, reflecting the constraint imposed by the shared wild-type, but paratope composition differs: AntiFold variants (Fig. 7A) recruit both chains comparably, the ProteinDPO candidate (Fig. 7C) is H-chain dominant, and SimBinder-IF—which yields the most Pareto-optimal variants (six; Fig. 7D)—spans the widest range of approach angles and H/L positioning.

Table 2 summarizes the interface interactions. SimBinder-IF achieves the strongest predicted binding (FoldX ΔΔG from −63.9 to −75.1\, kcal/mol vs.\-48.5 to -61.3 for AntiFold, −47.8 to − 53.1 for ESM-IF, and-52.0 for ProteinDPO), with diverse interaction profiles—hydrophobic-rich variants (6331, 6721) and a salt-bridge-rich variant (7323). ESM-IF shows the lowest ipLDDT (48–73), while AntiFold variant 1178 attains the highest interface confidence (iPTM 0.84, ipLDDT 87.7).

**Table 2:** Binding interface interaction statistics for all Pareto-optimal variants.

Method	Variant ID	HB	SB	HC	**C** π	PP	**FoldX** ΔΔG **(kcal/mol)**	iPTM	ipLDDT
AntiFold	30	9	4	191	2	0	−61.32	0.37	73.28
AntiFold	384	4	2	80	1	0	−48.47	0.44	78.16
AntiFold	1083	7	1	55	3	0	−59.27	0.35	80.89
AntiFold	1178	12	2	25	4	0	−60.88	0.84	87.72
ESM-IF	1768	2	2	57	2	0	−53.06	0.36	48.47
ESM-IF	2708	6	0	116	5	0	−49.39	0.36	51.64
ESM-IF	2800	5	2	98	0	0	−47.82	0.35	48.23
ESM-IF	2957	7	0	88	6	0	−48.81	0.44	73.11
ProteinDPO	3588	11	2	106	1	0	−51.96	0.51	83.03
SimBinder-IF	6132	7	2	114	2	0	−75.13	0.36	76.27
SimBinder-IF	6331	12	3	173	2	1	−68.35	0.51	75.03
SimBinder-IF	6465	9	2	60	6	0	−63.92	0.43	73.12
SimBinder-IF	6649	5	1	82	4	0	−64.46	0.38	74.77
SimBinder-IF	6721	9	3	169	1	0	−71.68	0.37	47.44
SimBinder-IF	7323	5	8	15	2	0	−67.83	0.35	70.85

Hydrogen bonds (HB), salt bridges (SB), hydrophobic contacts (HC), cation–π interactions (Cπ), and π–π stacking (PP) were computed from AlphaFold 3 predicted complex structures using PLIP. Binding ΔΔG (kcal/mol) was predicted by FoldX; iPTM and ipLDDT are AlphaFold 3 interface confidence metrics.

## 4 Discussion

Our study establishes an effective training framework for antibody affinity optimization that goes beyond conventional preference learning. We demonstrate that the combination of Simple Preference Optimization (SimPO) with a frozen structure encoder is crucial for achieving superior performance, rather than preference optimization alone. By keeping the encoder parameters fixed, we preserve the robust structural representations learned during pre-training, while the targeted fine-tuning of the decoder effectively aligns the generative likelihood with binding affinity. This strategy proves to be training-efficient yet highly effective, enabling SimBinder-IF to outperform baselines that employ full-parameter fine-tuning or alternative preference optimization objectives like DPO.

Critically, SimBinder-IF preserves generic protein–protein interaction modelling in a class-dependent manner: performance improves for AB/AG and Pr/PI complexes, but the aggregate means remain slightly below ESM-IF and ProteinDPO because of trade-offs in TCR/pMHC and heterogeneous ‘Other’ complexes.

Our results reveal that SimBinder-IF’s gains stem primarily from enhanced interface-specific modelling rather than global stability assessment. This focus on binding interfaces—where antibody-antigen recognition occurs—enables the model to prioritize functionally relevant epitopes critical for therapeutic antibody design. The ability to distinguish immunodominant epitopes and predict favourable engagement profiles has direct translational implications, as effective antibodies must target accessible and conserved regions to minimize viral escape or off-target effects.

We believe ρ≈0.32 is practically meaningful for candidate prioritization: Shanker *et al*. (2024) showed that ESM-IF1—our baseline at ρ≈0.26—was already sufficient to yield up to 25-fold improvement in neutralization and 37-fold improvement in affinity in a real antibody engineering campaign, and experimental label noise imposes a practical ceiling on achievable ρ well below 1.0.

Despite these promising results, several limitations remain. The current reward function is single-objective, optimizing solely for binding affinity. While our variants show good structural metrics in silico, we do not explicitly model other critical developability attributes such as immunogenicity, aggregation propensity (viscosity), or chemical stability. Additionally, the reliance on in-silico proxies like ΔΔG and AlphaFold structure prediction serves as a necessary surrogate for high-throughput validation but cannot fully replace experimental characterization. Moreover, while freezing the encoder promotes efficiency, it inherently limits the model’s ability to account for significant backbone conformational changes that might be induced by certain mutations, potentially capping performance where flexibility is key. Finally, the case study evaluation carries a degree of metric circularity: Stage 1 of the screening pipeline filters by FoldX ΔΔG, the same metric highlighted as the primary advantage in the Pareto-front analysis.

Looking forward, future iterations of SimBinder-IF could integrate multi-objective preference optimization to simultaneously balance affinity with developability constraints, potentially leveraging thermodynamic ensemble representations as demonstrated by AbLEF (Rollins *et al*. 2024) to capture dynamic molecular behaviour. To address backbone flexibility, we envision exploring semi-frozen strategies where specific layers of the encoder are fine-tuned, or incorporating SE(3)-equivariant modules that can dynamically adjust to induced fit effects. Coupling this computational framework with active learning loops driven by high-throughput wet-lab screening would further refine the reward model, paving the way for a truly comprehensive, data-driven platform for therapeutic antibody discovery.

## Supplementary Material

btag548_Supplementary_Data

## Data Availability

The source code and model weights for SimBinder-IF are available at https://github.com/MSBMI-SAFE/SimBinder-IF. The archived code is available at Zenodo (DOI: https://doi.org/10.5281/zenodo.20496471; record: https://zenodo.org/records/20496471). The datasets analyzed in this study are publicly available as follows: AbBiBench at https://huggingface.co/datasets/AbBibench/Antibody_Binding_Benchmark_Dataset; SKEMPI 2.0 at https://life.bsc.es/pid/skempi2; and the m396 SARS-CoV-2 mutational dataset from Desautels *et al*. (2020) at https://doi.org/10.1101/2020.04.03.024885.
