# Supplementary material for: SimBinder-IF: structure-aware antibody affinity optimization via efficient preference learning
Source: Bioinformatics. 2026 Jul 24;42(10):btag548. doi: 10.1093/bioinformatics/btag548 (PMC13689999; doi:10.1093/bioinformatics/btag548)
Supplement: Supplementary file 1 [file btag548_supplementary_data.pdf]

Supplementary Material

1 Downstream Residue-Level Classification Task: Paratope Prediction

To verify that SimBinder-IF retains the robust single-residue embedding capability of the original Evolutionary Scale Modeling inverse folding (ESM-IF) model, we evaluated it on a downstream residue-level classification task: paratope prediction. This task involves identifying the specific amino acid residues on an antibody's surface that interact with an antigen, serving as a direct probe of the model's ability to capture local functional features. We utilized the Therapeutics Data Commons (TDC) Paratope dataset[1] (SAbDab Liberis split), a rigorous benchmark of curated antibody-antigen complexes consisting of 716 training, 102 validation, and 205 test samples.

We compared the performance of SimBinder-IF against ESM-IF by freezing the embedding model and training a lightweight classifier on top of their respective structure-aware embeddings. SimBinder-IF achieved an area under the receiver operating characteristic curve (ROC-AUC) of 0.791, slightly above ESM-IF (0.783; Figure S1a). As shown in the precision-recall (PR) plot (Fig. S1b), the baseline ESM-IF model achieves an Average Precision (AP) of 0.207, slightly outperforming SimBinder-IF (AP = 0.196) in the high-precision regime (low recall). Thus, the small ROC-AUC increase and small AP decrease indicate that residue-level classification performance is broadly preserved rather than uniformly improved after fine-tuning.

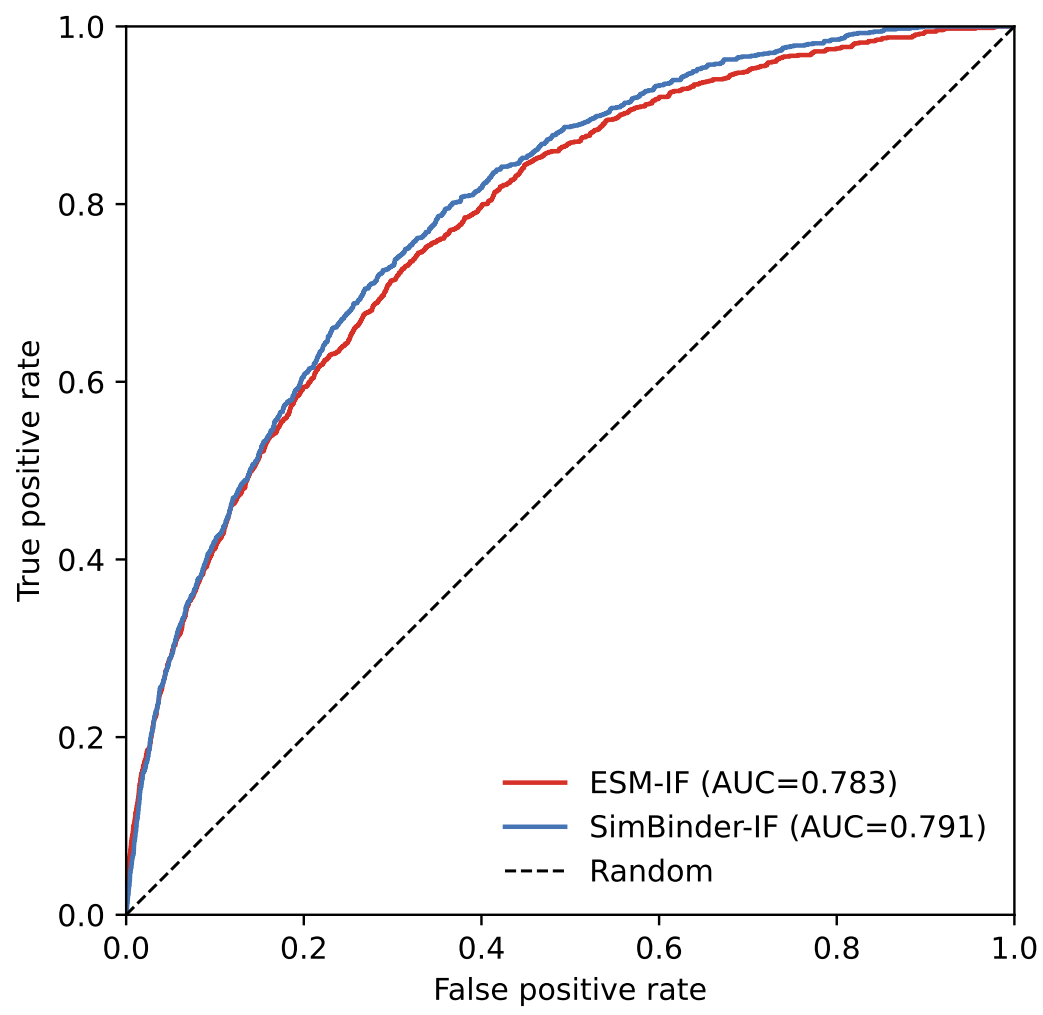

(a) ROC Curves

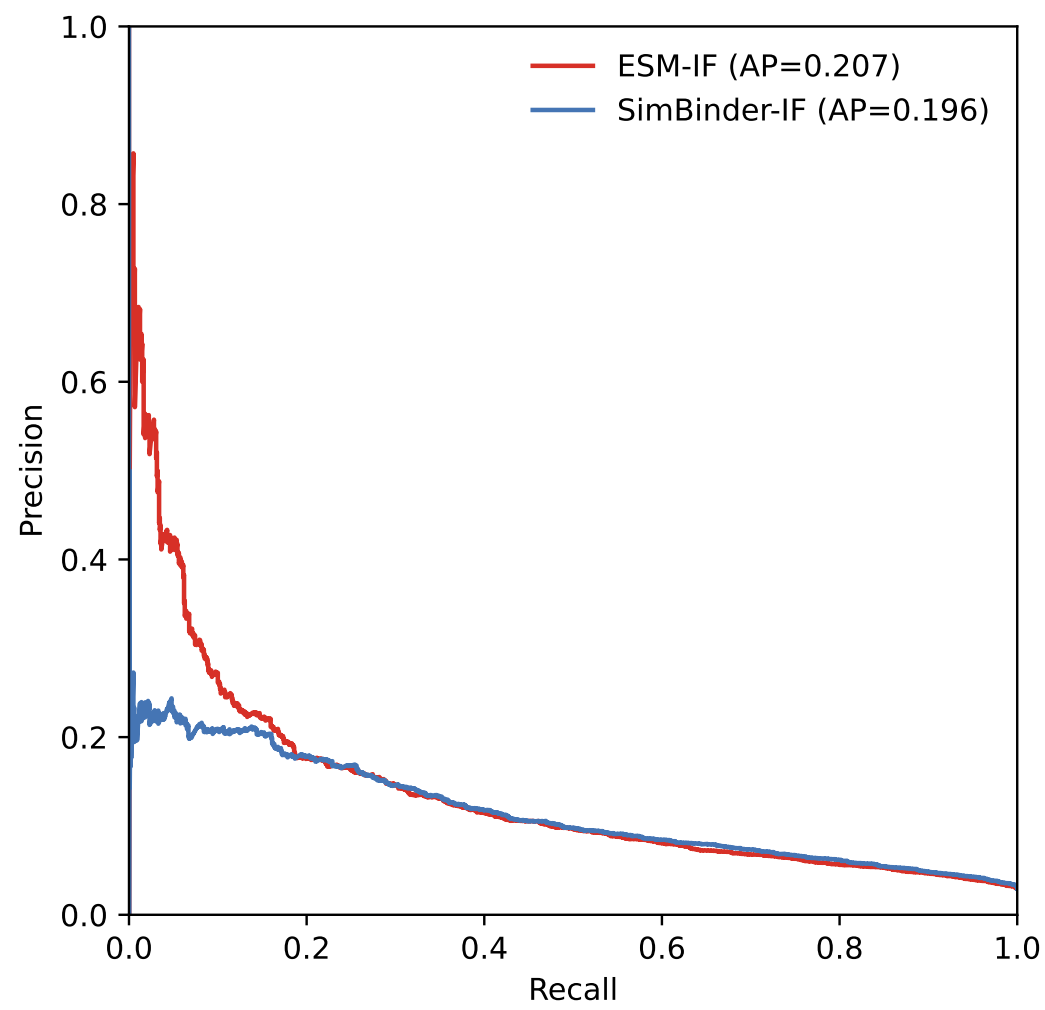

(b) PR Curves

Figure S1: Performance comparison on TDC Paratope Prediction task.

2 Bootstrap Confidence Intervals and Paired Statistical Tests for AbBiBench Results

Table S1 reports per-assay Spearman correlations with 95% confidence intervals for all comparison models across the seven held-out AbBiBench test assays. Table S2 presents the 10-fold improvement@10 and 20-fold improvement@20 proportions across the five K_d-based assays (excluding 5a12_ang2 and 1n8z, which

[1] `https://tdcommons.ai/single_pred_tasks/paratope/`

are incompatible with fold-change computation). For rank-based continuous metrics (Spearman correlation), 95% confidence intervals (CIs) were estimated using bootstrapping (5,000 resamples). Due to the small fixed denominator and discrete nature of the Top-N proportion metrics (n-fold improvement@N), we report only point estimates alongside means and standard deviations across random seeds.

To assess the sensitivity to the training random seed, we additionally tested non-baseline models trained with seeds 2026 and 3407. The per-assay metrics for these seeds are included as sub-rows in Tables S1 and S2, with the main rows reporting the three-seed averages. The performance is highly stable across different seeds. For Spearman ρ, the results are: SimBinder-IF (0.323 ± 0.012), ESM-IF-DPO-Dec (0.293 ± 0.007), ESM-IF-SimPO-Hybrid (0.267 ± 0.007), and ESM-IF-SimPO-All (0.264 ± 0.005). For 10-fold improvement@10, the results are: SimBinder-IF (0.487 ± 0.031), ESM-IF-DPO-Dec (0.420 ± 0.020), ESM-IF-SimPO-Hybrid (0.393 ± 0.012), and ESM-IF-SimPO-All (0.400 ± 0.040). For 20-fold improvement@20, the results are: SimBinder-IF (0.453 ± 0.012), ESM-IF-DPO-Dec (0.427 ± 0.006), ESM-IF-SimPO-Hybrid (0.310 ± 0.061), and ESM-IF-SimPO-All (0.333 ± 0.032).

In addition to estimating robust confidence intervals on each individual assay, we conducted a Wilcoxon signed-rank test across the seven test complexes to evaluate macro-level statistical significance. SimBinder-IF outperformed ESM-IF on 5 out of 7 assays based on the three-seed averages; however, this overall gain yielded $p = 0.188$, which is not statistically significant. We note that the small sample size ($n = 7$) intrinsically limits the statistical power of the Wilcoxon test, preventing a p-value below 0.05 unless performance improvements occur almost uniformly across all datasets. Therefore, the bootstrap CIs and multi-seed replications provide a more sensitive and informative measure of model reliability in this setting.

Table S1: **Per-assay Spearman ρ [95% bootstrap CI] for all comparison models.** CIs computed via 5,000 resamples. Bold indicates the best value per assay column among main model rows. The SimBinder-IF, ESM-IF-DPO-Dec, ESM-IF-SimPO-Hybrid, and ESM-IF-SimPO-All rows report the mean ρ across three random seeds (seed42, seed2026, seed3407); per-seed results with 95% bootstrap CIs are shown in the indented sub-rows below.

**Model**	**4fqi_h3**	**3gbn_h9**	**aayl49**	**aayl49_ML**	**aayl50**	**1n8z**	**5a12_ang2**	**Mean ρ**
ESM-IF	0.494 [0.488, 0.500]	0.540 [0.507, 0.571]	0.387 [0.362, 0.412]	0.266 [0.247, 0.285]	0.138 [0.120, 0.156]	−0.108 [−0.204, −0.012]	0.130 [0.067, 0.192]	0.264
AntiFold	0.374 [0.368, 0.381]	0.272 [0.229, 0.314]	**0.395** [0.369, 0.420]	0.136 [0.115, 0.156]	0.073 [0.055, 0.091]	0.161 [0.069, 0.251]	**0.177** [0.114, 0.238]	0.227
ProteinDPO	0.491 [0.485, 0.496]	0.625 [0.596, 0.652]	0.353 [0.327, 0.379]	0.148 [0.127, 0.168]	−0.052 [−0.070, −0.034]	−0.075 [−0.173, 0.024]	0.086 [0.022, 0.149]	0.225
Diffab	−0.007 [−0.015, 0.001]	0.614 [0.585, 0.642]	0.149 [0.120, 0.178]	0.002 [−0.018, 0.023]	0.018 [−0.000, 0.036]	−0.023 [−0.112, 0.069]	0.027 [−0.037, 0.091]	0.111
SaProt	0.279 [0.272, 0.286]	0.601 [0.571, 0.629]	−0.269 [−0.296, −0.241]	0.107 [0.086, 0.127]	−0.110 [−0.128, −0.092]	0.115 [0.023, 0.206]	−0.083 [−0.146, −0.020]	0.091
CurrAb	0.134 [0.126, 0.141]	0.228 [0.184, 0.271]	0.028 [−0.002, 0.058]	0.197 [0.177, 0.217]	0.007 [−0.012, 0.025]	−0.387 [−0.466, −0.300]	0.051 [−0.013, 0.115]	0.037
ESM-2	−0.017 [−0.025, −0.009]	0.380 [0.340, 0.419]	−0.037 [−0.067, −0.007]	−0.136 [−0.156, −0.116]	−0.028 [−0.046, −0.010]	−0.131 [−0.220, −0.039]	−0.072 [−0.135, −0.008]	−0.006
dyMEAN	0.021 [0.013, 0.029]	−0.024 [−0.069, 0.022]	−0.030 [−0.060, −0.001]	−0.010 [−0.030, 0.011]	−0.015 [−0.033, 0.003]	−0.003 [−0.100, 0.095]	0.010 [−0.054, 0.074]	−0.007
SimBinder-IF	0.532	0.578	0.369	**0.300**	0.139	0.273	0.071	**0.323**
seed42	0.627 [0.623, 0.632]	0.612 [0.582, 0.639]	0.378 [0.353, 0.404]	0.314 [0.295, 0.332]	0.139 [0.121, 0.157]	0.116 [0.020, 0.208]	0.053 [−0.011, 0.116]	0.320
seed2026	0.503 [0.497, 0.509]	0.554 [0.520, 0.585]	0.343 [0.317, 0.370]	0.273 [0.254, 0.292]	0.134 [0.116, 0.153]	0.328 [0.240, 0.415]	0.053 [−0.011, 0.116]	0.313
seed3407	0.465 [0.458, 0.471]	0.569 [0.546, 0.591]	0.387 [0.362, 0.412]	0.312 [0.293, 0.331]	0.144 [0.126, 0.163]	0.375 [0.289, 0.457]	0.107 [0.043, 0.169]	0.337
ESM-IF-DPO-Dec	0.582	0.520	0.252	0.192	0.031	0.351	0.121	0.293
seed42	0.615 [0.610, 0.619]	0.490 [0.454, 0.524]	0.261 [0.233, 0.289]	0.189 [0.169, 0.209]	0.016 [−0.002, 0.034]	0.349 [0.260, 0.433]	0.134 [0.071, 0.196]	0.293
seed2026	0.575 [0.568, 0.580]	0.576 [0.547, 0.622]	0.260 [0.224, 0.296]	0.210 [0.190, 0.222]	0.037 [0.017, 0.052]	0.321 [0.226, 0.415]	0.112 [0.054, 0.150]	0.299
seed3407	0.556 [0.550, 0.562]	0.495 [0.474, 0.524]	0.235 [0.210, 0.256]	0.176 [0.150, 0.192]	0.040 [0.024, 0.065]	0.384 [0.297, 0.495]	0.118 [0.055, 0.160]	0.286
ESM-IF-SimPO-Hybrid	0.576	**0.699**	0.124	−0.092	0.182	0.286	0.096	0.267
seed42	0.570 [0.565, 0.575]	0.717 [0.694, 0.739]	0.165 [0.136, 0.194]	−0.149 [−0.169, −0.129]	0.273 [0.256, 0.290]	0.220 [0.128, 0.309]	0.112 [0.049, 0.175]	0.273
seed2026	0.554 [0.547, 0.559]	0.685 [0.658, 0.704]	0.162 [0.134, 0.209]	−0.120 [−0.137, −0.091]	0.250 [0.238, 0.263]	0.241 [0.155, 0.353]	0.113 [0.066, 0.161]	0.269
seed3407	0.604 [0.599, 0.609]	0.696 [0.678, 0.713]	0.044 [0.014, 0.074]	−0.008 [−0.029, 0.013]	0.023 [0.005, 0.041]	0.397 [0.315, 0.475]	0.064 [0.000, 0.127]	0.260
ESM-IF-SimPO-All	0.430	0.440	0.091	0.172	0.255	**0.469**	−0.008	0.264
seed42	0.433 [0.426, 0.439]	0.440 [0.403, 0.476]	0.098 [0.069, 0.128]	0.158 [0.138, 0.178]	0.235 [0.218, 0.253]	0.456 [0.376, 0.533]	−0.008 [−0.072, 0.056]	0.259
seed2026	0.426 [0.418, 0.434]	0.408 [0.372, 0.450]	0.106 [0.088, 0.141]	0.174 [0.151, 0.198]	0.269 [0.251, 0.284]	0.462 [0.398, 0.543]	0.011 [−0.086, 0.068]	0.265
seed3407	0.430 [0.423, 0.436]	0.473 [0.431, 0.517]	0.070 [0.029, 0.096]	0.185 [0.165, 0.211]	0.260 [0.249, 0.281]	0.489 [0.406, 0.587]	−0.027 [−0.078, 0.064]	0.269

3 Pairwise Sequence Identity Between Training and Test Complexes

Figure S2 shows the pairwise sequence identity between all training and test complexes used in the antigen-split evaluation.

Antigen sequence identity. For the vast majority of training–test pairs, antigen sequence identity is below 4%, confirming that the test antigens have no close sequence homologs among the training antigens. The exception is the `aayl49/aayl49_ML/aayl50` test group paired with `aayl51/aayl52` training complexes, where the SARS-CoV-2 Spike HR2 antigen is shared (100% identity); however, the antibody sequences and experimental affinity assays differ, so these pairs still constitute a meaningful generalization challenge. Overall, the antigen-split evaluation ensures that observed performance gains reflect generalization to sequentially different antigens rather than interpolation from training data.

Antibody (VH+VL) sequence identity. Excluding same-scaffold pairs (3gbn h1/h9 and 4fqi h1/h3, which share the identical PDB-derived antibody sequence), the mean antibody (VH+VL) sequence iden-

Table S2: **Per-assay 10-fold improvement@10 and 20-fold improvement@20 proportions for all comparison models.** Proportions of the top-10 ranked variants achieving ≥10-fold affinity improvement and top-20 ranked variants achieving ≥20-fold affinity improvement over the wild type. Evaluated on 5 K_d-based assays; 5a12_ang2 and 1n8z excluded. The SimBinder-IF, ESM-IF-DPO-Dec, ESM-IF-SimPO-Hybrid, and ESM-IF-SimPO-All rows report the mean proportion across three random seeds; per-seed results are shown in the indented sub-rows.

**Model**	**4fqi_h3**	**3gbn_h9**	**aayl49**	**aayl49_ML**	**aayl50**	**Mean**
**Panel A: 10-fold improvement@10**						
ESM-IF	1.000	1.000	0.000	0.600	0.000	**0.520**
ProteinDPO	0.700	1.000	0.000	0.300	0.000	0.400
AntiFold	0.000	1.000	0.100	0.500	0.000	0.320
CurrAb	0.200	0.900	0.000	0.100	0.100	0.260
Diffab	0.100	1.000	0.000	0.200	0.000	0.260
SaProt	0.100	1.000	0.000	0.000	0.100	0.240
ESM-2	0.000	1.000	0.000	0.100	0.000	0.220
dyMEAN	0.100	0.800	0.000	0.000	0.000	0.180
SimBinder-IF	1.000	1.000	0.033	0.333	0.067	0.487
seed42	1.000	1.000	0.100	0.400	0.100	0.520
seed2026	1.000	1.000	0.000	0.300	0.000	0.460
seed3407	1.000	1.000	0.000	0.300	0.100	0.480
ESM-IF-DPO-Dec	0.867	0.967	0.000	0.200	0.067	0.420
seed42	0.800	0.900	0.000	0.200	0.100	0.400
seed2026	0.800	1.000	0.000	0.300	0.100	0.440
seed3407	1.000	1.000	0.000	0.100	0.000	0.420
ESM-IF-SimPO-All	0.467	0.933	0.067	0.367	0.167	0.400
seed42	0.400	1.000	0.100	0.300	0.200	0.400
seed2026	0.600	0.900	0.100	0.400	0.200	0.440
seed3407	0.400	0.900	0.000	0.400	0.100	0.360
ESM-IF-SimPO-Hybrid	0.967	1.000	0.000	0.000	0.000	0.393
seed42	0.900	1.000	0.000	0.000	0.000	0.380
seed2026	1.000	1.000	0.000	0.000	0.000	0.400
seed3407	1.000	1.000	0.000	0.000	0.000	0.400
**Panel B: 20-fold improvement@20**						
ESM-IF	0.850	1.000	0.050	0.250	0.000	0.430
ProteinDPO	0.250	1.000	0.000	0.150	0.000	0.280
Diffab	0.100	1.000	0.000	0.150	0.000	0.250
SaProt	0.150	1.000	0.000	0.000	0.050	0.240
AntiFold	0.000	1.000	0.050	0.100	0.000	0.230
ESM-2	0.000	1.000	0.000	0.050	0.000	0.210
dyMEAN	0.100	0.750	0.000	0.150	0.000	0.200
CurrAb	0.000	0.650	0.000	0.100	0.000	0.150
SimBinder-IF	0.933	1.000	0.067	0.250	0.017	**0.453**
seed42	0.850	1.000	0.100	0.300	0.050	0.460
seed2026	0.950	1.000	0.050	0.200	0.000	0.440
seed3407	1.000	1.000	0.050	0.250	0.000	0.460
ESM-IF-DPO-Dec	0.950	0.983	0.033	0.117	0.050	0.427
seed42	1.000	1.000	0.000	0.100	0.000	0.420
seed2026	0.900	0.950	0.100	0.100	0.100	0.430
seed3407	0.950	1.000	0.000	0.150	0.050	0.430
ESM-IF-SimPO-All	0.317	0.983	0.033	0.200	0.133	0.333
seed42	0.300	1.000	0.000	0.200	0.100	0.320
seed2026	0.400	0.950	0.000	0.300	0.200	0.370
seed3407	0.250	1.000	0.100	0.100	0.100	0.310
ESM-IF-SimPO-Hybrid	0.517	1.000	0.017	0.017	0.000	0.310
seed42	0.350	1.000	0.000	0.000	0.000	0.270
seed2026	0.300	1.000	0.050	0.050	0.000	0.280
seed3407	0.900	1.000	0.000	0.000	0.000	0.380

Table S3: **Paired Wilcoxon signed-rank test: SimBinder-IF vs. each baseline (Spearman ρ, 7 assays).** W: Wilcoxon statistic (sum of ranks of positive differences); maximum $W = 28$ at $n = 7$. p: one-sided p-value. Mean $\Delta\rho$, Wins/7, and W reflect values computed from the three-seed average correlations for fine-tuned models.

**Baseline**	**Mean ρ (baseline)**	**Mean $\Delta\rho$**	**Wins / 7**	W	**p-value**
ESM-IF	0.264	+0.059	5/7	20.0	0.188
ProteinDPO	0.225	+0.098	5/7	23.0	0.078
SaProt	0.091	+0.232	7/7	28.0	0.008^{**}
AntiFold	0.227	+0.096	4/7	22.0	0.109
CurrAb	0.037	+0.286	7/7	28.0	0.008^{**}
ESM-2	−0.006	+0.329	7/7	28.0	0.008^{**}
Diffab	0.111	+0.212	6/7	27.0	0.016^{*}
dyMEAN	−0.007	+0.330	7/7	28.0	0.008^{**}
ESM-IF-DPO-Dec	0.293	+0.030	4/7	21.0	0.148
ESM-IF-SimPO-All	0.264	+0.059	5/7	19.0	0.234
ESM-IF-SimPO-Hybrid	0.267	+0.056	2/7	13.0	0.578

** $p < 0.01$; * $p < 0.05$. Minimum achievable one-sided p at $n = 7$ is 0.0078.

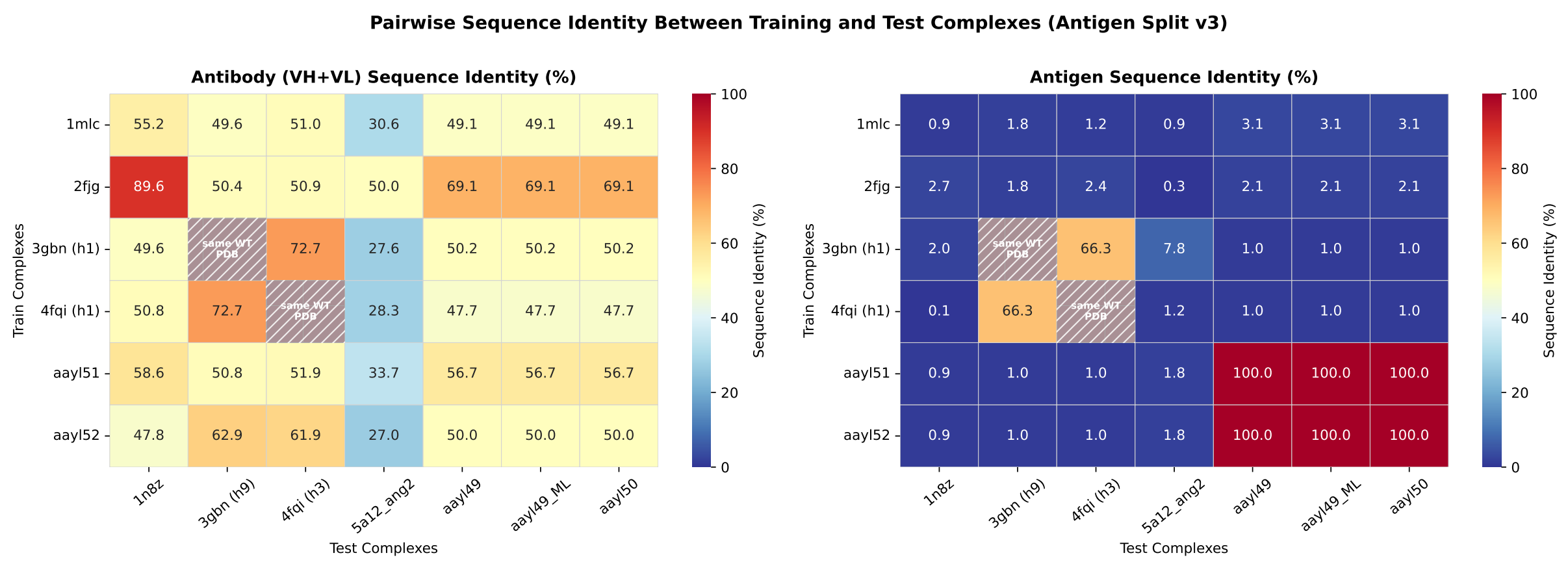

Figure S2: Pairwise sequence identity between training and test complexes (Antigen Split v3). Left: antibody (VH+VL) pairwise sequence identity (%). Right: antigen pairwise sequence identity (%). Hatched cells indicate same-scaffold pairs sharing the same wild-type PDB file (3gbn h1/h9 and 4fqi h1/h3), which differ only in the hemagglutinin subtype of the binding assay. Colour scale: blue (0%) to red (100%).

tity across all training–test pairs is approximately 53.2%. This is consistent with the natural conservation of immunoglobulin framework regions (typically 70–85% identity), while CDR loops—which determine antigen specificity—differ substantially, confirming that the test antibodies are distinct from those seen during training.

Cross-subtype generalization: 3gbn and 4fqi. The `3gbn_h1` (training) / `3gbn_h9` (test) pair and the `4fqi_h1` (training) / `4fqi_h3` (test) pair each share the same source PDB structure, so the antibody and antigen three-dimensional coordinates are identical at input. The distinction lies in the binding experiments: each pair measures affinity against a different influenza hemagglutinin (HA) subtype—H1 versus H9, and H1 versus H3, respectively—using independently collected $-\log K_d$ measurements. Despite near-complete variant overlap (1,812/1,887 $\approx 96\%$ for 3gbn; 65 093/65 094 $\approx 100\%$ for 4fqi), the binding score landscapes diverge substantially across subtypes. For 3gbn, the Spearman correlation between H1 and H9 binding scores is $\rho = 0.725$ (standard deviation of score differences = 0.70), indicating a meaningful reordering of variant rankings. For 4fqi, the correlation drops to $\rho = 0.379$ with a systematic offset of −2.935 kcal/mol (H3 affinity is uniformly lower than H1), meaning that H1-trained rankings explain only $\approx 14\%$ of the variance in H3 rankings. These cross-subtype pairs therefore represent a qualitatively distinct generalization challenge: given identical structural input, the model must capture features that are predictive of binding across divergent hemagglutinin subtypes rather than memorizing a single-subtype affinity landscape.

4 Training Hyperparameters and Training Efficiency Comparison

SimBinder-IF freezes the geometric vector perceptron graph neural network (GVP-GNN) structure encoder and fine-tunes only the sequence decoder. We use AdamW (learning rate 10^{-4}; batch size 32) for three epochs. For SimPO, the reward scaling factor is $\beta = 0.1$ and the margin is $\gamma = 0.1$. We select the checkpoint with the highest mean validation Spearman ρ, evaluated every 100 steps. All experiments use one NVIDIA H200 graphics processing unit (GPU). Figure S3 reports training time and peak GPU memory.

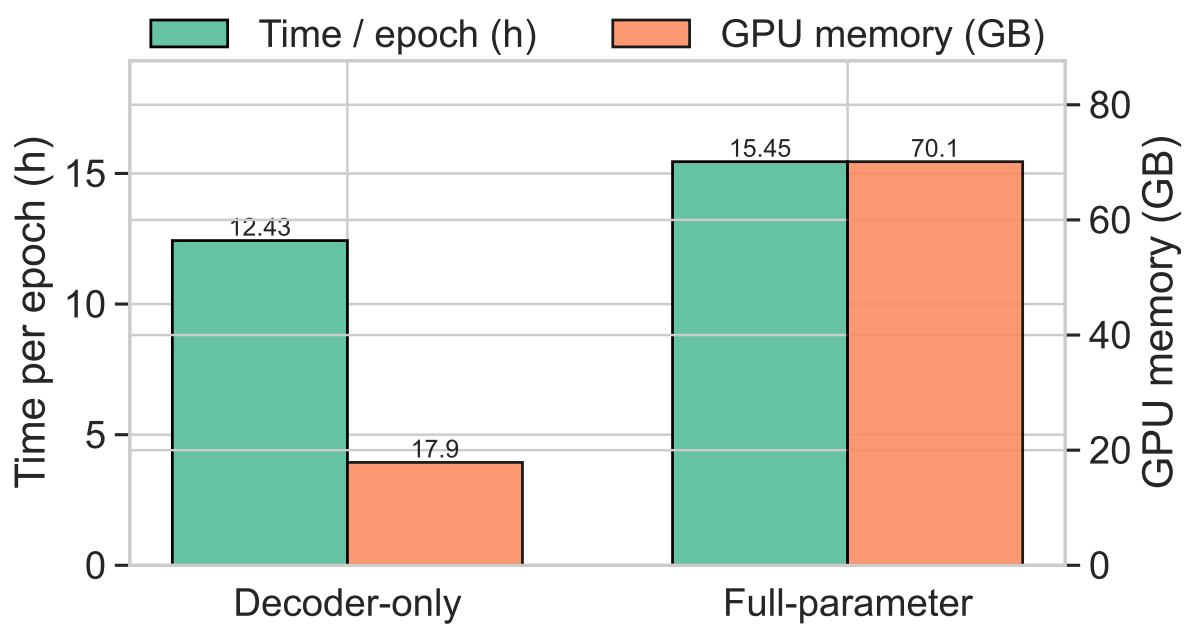

Figure S3: **Training efficiency comparison between decoder-only and full-parameter SimPO fine-tuning variants.** Comparison in terms of training time and GPU memory usage.

5 Recap of ESM Inverse Folding

The inverse folding problem involves inferring, from a fixed three-dimensional backbone conformation, the amino acid sequence most likely to fold into and stabilize that structure. In our previous work, Zhao *et al.* (2025), we benchmarked various protein modelling approaches and find that inverse folding methods—including ProteinMPNN (Dauparas *et al.* 2022), ESM-IF, and AntiFold (Høie *et al.* 2025)—outperform other model classes in the antibody design task. ESM-IF, as a representative inverse-folding model, is a graph-based transformer trained to predict amino acid identities from 3D backbone coordinates. Its architecture comprises:

- An invariant structural encoder f_{enc} that embeds atomic positions and pairwise geometric features into residue-level embeddings

$$\mathbf{E} = f_{\text{enc}}(X) \in \mathbb{R}^{n \times d}, \quad X = \{\mathbf{x}_i\}_{i=1}^{3n}.$$

- A sequence tokenizer f_{tok} maps each previously generated residue $y_{<i}$ into a learned embedding, which is concatenated with the structural embedding $\mathbf{E}$ to form the decoder input. An autoregressive decoder f_{dec} predicts the next residue conditioned on both the structure and the partial sequence:

$$p(Y \mid X) = \prod_{i=1}^{n} p\big(y_i \mid y_{<i}, \mathbf{E}\big),$$
$$p\big(y_i \mid y_{<i}, \mathbf{E}\big) = \text{softmax}\big(W\,h_i + b\big),$$
$$h_i = f_{\text{dec}}(\mathbf{E}, y_{<i}) \in \mathbb{R}^d, \quad Y = (y_1, \ldots, y_n).$$

During training, the model minimizes the negative log-likelihood

$$\mathcal{L}_{\text{NLL}} = -\sum_{(X,Y)} \log p(Y \mid X)$$

to recover native sequences. Although originally designed for sequence recovery, ESM-IF can be reinterpreted as a structure-conditioned generative model: given the 3D coordinates of a wild-type antibody–antigen complex, novel sequence variants are sampled by drawing from $p(Y \mid X)$ guided by the decoder's log-likelihood scores.

References

Dauparas J *et al.* Robust deep learning–based protein sequence design using ProteinMPNN. *Science* 2022;**378**:49–56.

Høie MH *et al.* AntiFold: improved structure-based antibody design using inverse folding. *Bioinform Adv* 2025;**5**:vbae202.

Zhao X *et al.* Benchmark for antibody binding affinity maturation and design. *arXiv preprint arXiv:2506.04235* 2025.
